# Long Term Outcomes in Living Kidney Donors Over 30 Years

**DOI:** 10.1016/j.ekir.2026.106690

**Published:** 2026-07-02

**Authors:** Joana Krättli, Deborah Buess, Marie Flohr, Matthias Diebold, Caroline Wehmeier, Daniel Sidler, Guido Beldi, Dela Golshayan, Tobias Zingg, Sophie de Seigneux, Fadi Haidar, Alexander Ritter, Isabelle Binet, Kerstin Hübel, Fabian Rössler, Jürg Steiger, Patricia Hirt-Minkowski

**Affiliations:** 1 Swiss Organ Living-Donor Health Registry (SOL-DHR), University Hospital Basel, Basel, Switzerland; 2 Department of Circulation, Thorax and Transplantation, Clinic for Transplantation Immunology and Nephrology, University Hospital Basel, Basel, Switzerland; 3 Department for Nephrology and Hypertension, University Hospital Insel, Berne, Switzerland; 4 Department of Visceral Surgery and Medicine, University Hospital Insel, Abdominal Center Berne, Switzerland; 5 Department for Medicine, Transplantation Center, Lausanne University Hospital (CHUV), Lausanne, Switzerland; 6 Department of Visceral and Transplant Surgery, Lausanne University Hospital (CHUV), Lausanne, Switzerland; 7 Nephrology and Hypertension Division, University Hospital Geneva (HUG), Geneva, Switzerland; 8 Clinic for Nephrology and Transplantation Medicine, HOCH Health Ostschweiz, Cantonal Hospital St. Gallen, St. Gallen, Switzerland; 9 Division of Nephrology, University Hospital Zurich, Zurich, Switzerland; 10 Department of Surgery and Transplantation, University Hospital Zurich, Zurich, Switzerland

**Keywords:** donor safety, kidney function decline, living kidney donation, living kidney donors, long-term outcomes, postdonation monitoring

## Abstract

**Introduction:**

Living kidney donor transplantation provides major benefits for recipients, but long-term donor safety remains a critical concern.

**Methods:**

We conducted a prospective, multicenter cohort study using data from the Swiss Organ Living-Donor Health Registry. A total of 1863 living kidney donors (1993–2018) with ≥ 5 years of follow-up were followed until death, kidney failure (KF), or last visit (censoring March 31, 2024). Kidney function trajectories from 12 months postdonation onward were analyzed using linear mixed-effects models. A composite secondary end point included early death, dialysis-dependent KF, estimated glomerular filtration rate (eGFR) < 30 ml/min per 1.73 m^2^, or persistent proteinuria. Risk factors were assessed.

**Results:**

A total of 1863 living kidney donors (median age 53 years, 66.2% female) were included; 15.5% had treated arterial hypertension before donation. Median predonation eGFR was 96 ml/min per 1.73 m^2^, 1% had an eGFR < 60 ml/min per 1.73 m^2^. Median follow-up was 12 years (interquartile range [IQR]: 7–18 years). Kidney function remained stable, with an average annual eGFR change of +0.14 ml/min per 1.73 m^2^. Slopes differed by donor characteristics, as follows: women, nonobese, and those without predonation hypertension presented with more stable slopes, whereas obese and hypertensive donors showed attenuated slopes. No differences were observed by age or predonation eGFR. Three donors (0.2%) progressed to dialysis-dependent KF. Overall, 17 donors (0.9%) developed an eGFR < 30 ml/min per 1.73 m^2^, 28 (1.5%) developed persistent severe proteinuria, and 40 (2.1%) reached the combined end point. Male sex, age, and predonation obesity independently predicted the combined endpoint.

**Conclusion:**

These findings support safety while underscoring the need for standardized long-term surveillance to optimize donor care and counseling.

## Graphical abstract

**Figure:**
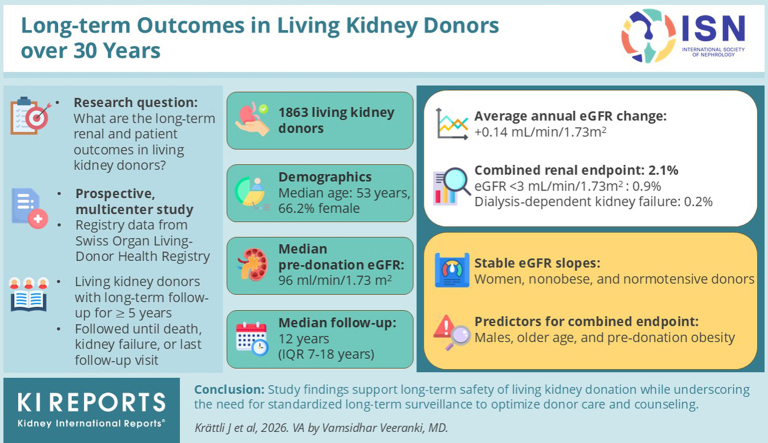

Living kidney donor transplantation is the optimal treatment for KF, increasing organ availability, improving graft and patient survival, and reducing healthcare costs and waiting time through preemptive transplantation.^1^, ^2^, ^3^, ^4^, ^5^, ^6^, ^7^ According to the Global Observatory on Donation and Transplantation, 111,135 kidney transplants were performed worldwide in 2023, with 39% from living donors.^8^ In Switzerland, living kidney donation (LKD) accounts for approximately one-third of all kidney transplants.^9^ Recipient benefits are well established, but donor safety remains paramount. Donor nephrectomy carries a low perioperative risk, making living donation ethically acceptable.^10^, ^11^, ^12^, ^13^, ^14^ However, long-term complications are less clearly characterized. Two cohort studies with median follow-up of 7.6 and 15.1 years reported an increased risk of KF in donors compared with matched healthy nondonors, although the absolute risk remained low.^15^^,^^16^ A recent systematic review and meta-analysis reported an approximately 5-fold increased risk of KF after LKD but identified moderate to critical risk of bias across included studies, highlighting important limitations of the available evidence.^17^ Other studies contributed to the identification of subgroups with a higher risk of KF.^18^, ^19^, ^20^ Most evidence is derived from studies of US donor populations, and the findings may not be directly transferable to European or Swiss settings because of differences in demographics and healthcare systems. Beyond this, the long-term evolution of kidney function after donation in prospectively followed cohorts remains incompletely understood.^21^, ^22^, ^23^, ^24^

Owing to global organ shortage, donor selection criteria have expanded to include older individuals and those with comorbidities.^25^, ^26^, ^27^ Accordingly, both the Kidney Disease: Improving Global Outcomes (KDIGO)^28^ and Swiss Academy of Medical Sciences^29^ guidelines accept donor candidates with well-controlled hypertension without end-organ damage. Donors show higher rates of hypertension and albuminuria in the first decade postnephrectomy.^30^, ^31^, ^32^, ^33^, ^34^ However, this may reflect genetic predisposition (since biologically-related donors are more likely than nondonors to have relatives with KF and/or hypertension) or closer follow-up rather than causal effects.^32^^,^^35^^,^^36^ A recently published prospective multicenter cohort study of living kidney donors and nondonors determined the risk of hypertension, albuminuria, and eGFR decline in 924 normotensive donors compared with 396 nondonors with similar baseline health and an identical follow-up schedule.^37^ In this study, the risks for hypertension and albuminuria were not significantly different in donors compared with controls.^37^ After the initial drop in eGFR from nephrectomy, donors had a slower mean rate of eGFR decline than nondonors but were more likely to experience an eGFR between 30 and 60 ml/min per 1.73 m^2^ at least once.^37^ A limitation was the short median follow-up of 7.3 years (IQR: 6.0–9.0 years),^37^ as certain risks associated with LKD may not manifest until beyond the first decade postdonation.^19^^,^^38^ This underscores the need for robust long-term data on living kidney donor outcomes to better inform clinical practice.

We therefore conducted a prospective national cohort study to characterize kidney function trajectories and identify risk factors for adverse outcomes. We hypothesized that long-term real-world data would enable early identification of donors at increased risk and support targeted postdonation surveillance and future donor selection.

## Methods

### Prospective Cohort Study and Donor Population

**Figure 1:**
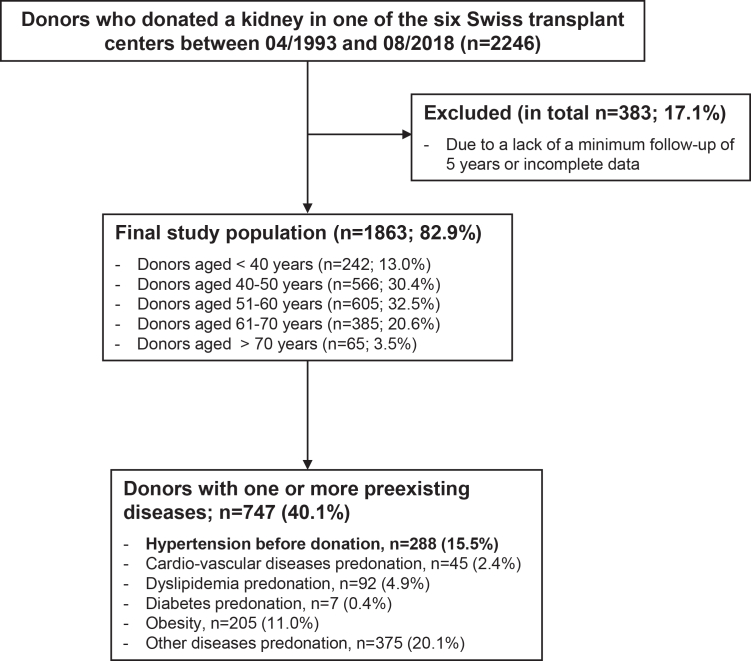
Study flowchart. Donors with a lack of a minimum follow-up of 5 years or incomplete data (*n* = 383) were excluded, thus the final population consisted of *n* = 1863 donors who donated a kidney between April 1993 and August 2018.

The Swiss Organ Living-Donor Health Registry, established in 1993 as the world’s first prospective registry, provides lifelong follow-up of all living kidney donors in Switzerland.^39^ In this study, we included all living kidney donors recorded in the national registry since 1993 who had a minimum follow-up of 5 years and at least 2 documented follow-up visits between 1 and 5 years after donation. The study was approved by the Ethics Committee of Northwestern and Central Switzerland (www.eknz.ch; project-ID 2023-02076). Between April 1993 and August 2018, 2246 individuals donated a kidney at 1 of the 6 Swiss transplant centers. Of these, 383 were excluded because they had < 5 years of follow-up or only 1 visit between 1 and 5 years postdonation. The final cohort included 1863 donors with complete data and documented informed consent. Outcomes were assessed until March 31, 2024 (Figure 1).

### Pre and Postdonation Management

The national registry schedules follow-up visits for living kidney donors at 1, 3, 5, 7, and 10 years postdonation, and every 2 years thereafter. Data collection before donation, at discharge, and during follow-up has been described in detail previously.^14^^,^^39^ Briefly, transplant centers inform donors about the registry and submit baseline data (demographics, comorbidities, and donor-recipient relationship) to Swiss Organ Living-Donor Health Registry. Follow-up data collected by physicians during follow-up visits included kidney function, metabolic parameters, medication use, and newly diagnosed complications. Office blood pressure is measured 3 times, and the mean value recorded.

### Data Collection

Baseline donor characteristics include sex, age, height, weight, body mass index (BMI), blood pressure, predonation medication, and comorbidities. Preexisting conditions were categorized as follows: (i) arterial hypertension, defined as the use of any antihypertensive medication before donation; (ii) cardiovascular disease, including coronary heart disease, aortic aneurysm, or cardiac arrhythmia (excluding hypertension); (iii) metabolic disorders, comprising obesity (body mass index [BMI] ≥ 30 kg/m^2^, according to the World Health Organization classification), type 2 diabetes mellitus (T2DM; hemoglobin A1c ≥ 6.5% or antidiabetic therapy described), and dyslipidemia treated with lipid-lowering agents; and (iv) other diseases, including pulmonary, rheumatic, musculoskeletal, endocrine, or neoplastic disorders. Postdonation conditions were classified analogously, except for hypertension—new-onset hypertension was recorded by the treating physician and confirmed by repeatedly elevated blood pressure readings (> 140/90 mm Hg), irrespective of antihypertensive treatment initiation.

Collected pre and postdonation laboratory parameters include serum creatinine, eGFR (calculated using the chronic kidney disease (CKD)-Epidemiology Collaboration 2009 formula) and urine analysis (i.e., urine protein-to-creatinine ratio [UPCR] and urine albumin-to-creatinine ratio [UACR], respectively). Laboratory parameters with a focus on metabolic values including hemoglobin A1c have only been collected in the national registry since the second half of 2017 and were discussed in detail elsewhere.^40^ Since 1993, blood and urine samples for all laboratory parameters have been sent to a core laboratory (Viollier AG, Basel, Switzerland).

### Objectives

The primary objective was to analyze long-term kidney function after LKD. All available eGFR measurements from 12 months after nephrectomy onward were used to account for changes caused by compensatory hyperfiltration of the remaining kidney after loss of nephron mass through nephrectomy within the first year postdonation. Donors were followed from the date of donation until death, initiation of dialysis because of KF, or the last regular follow-up visit, with follow-up continuing until March 31, 2024.

Furthermore, a combined secondary endpoint was prespecified, comprising early death after LKD (within 6 months postdonation), initiation of dialysis because of KF, decline of eGFR to < 30 ml/min per 1.73 m^2^, or persistent severe proteinuria, defined as a UPCR > 50 mg/mmol and/or UACR > 30 mg/mmol on at least 2 consecutive follow-up visits (according to KDIGO guidelines).^41^ The secondary endpoint was end pointfree donor survival. Additional analyses were performed to identify baseline risk factors associated with unfavorable outcomes.

### Statistical Analysis

Statistical analyses were performed using JMP Student Version 18.0 (SAS Institute Inc., Cary, NC) and R version 4.4.1 (R Foundation for Statistical Computing, Vienna, Austria). Categorical data were summarized as counts and percentages and analyzed using Fisher’s exact test or Pearson’s chi-squared test, as appropriate. Continuous variables were summarized as medians with IQR and compared using the Wilcoxon signed-rank test. No sample size calculation was performed, as this was a retrospective analysis. Diluted urine samples with creatinine values < 3 mmol/l were excluded from UPCR and UACR analyses.

We used a linear mixed-effects model to analyze longitudinal changes in eGFR over time. As the decrease in kidney function between predonation and 1 year postdonation is expected because of nephron loss, analyses were restricted to eGFR measurements from 1 year onward. In this model, time was treated as a continuous variable to capture the trajectory of eGFR. Fixed effects included time and its interactions with obesity status, sex, and donor age at donation. To account for potential nonlinear associations between baseline eGFR and subsequent trajectories, natural cubic splines with 3 degrees of freedom were applied and interacted with time. Random intercepts and slopes for time were included at the subject level to allow individual variation in both baseline eGFR and its rate of change. Model fitting was performed using the *lmer* function (lme4 package, R) with maximum likelihood estimation and a robust optimizer. Inference on time slopes (“rates of change”) used estimated marginal trends, averaging over other covariates in proportion to their observed distribution. To assess robustness of slope estimates, individual influence was examined. Random slopes were screened for extreme values relative to their standard errors and for participants with a high number of creatinine measurements. A sensitivity analysis excluding the most influential cases did not materially change the fixed-effect estimates, indicating that results were not driven by a few individuals. Because of many missing values, the association of predonation hypertension with the longitudinal slope of kidney function after LKD was analyzed in a separate model.

Endpoint-free donor survival was analyzed by the Kaplan–Meier method, and groups were compared using the log-rank test. Donors were stratified by whether they reached the combined endpoint postdonation. Multivariable Cox proportional hazards regression was performed to adjust for confounders. Predictors were selected based on information available at donation (sex, age, predonation arterial hypertension, and obesity) without *P*-value thresholds or automated selection. The 1:10 rule-of-thumb was applied, including 1 variable per 10 events to reduce overfitting. To account for the long observation time and potential changes in follow-up after LKD, the donation era was included as a prespecified confounder. Final models included sex, age, era of donation, and predonation obesity and arterial hypertension. Statistical significance was set at a 2-tailed *P* < 0.05. Missing values were not imputed.

## Results

### Baseline Characteristics

Baseline characteristics of individuals who donated a kidney between April 1993 and August 2018 are shown in Figure 1 and summarized in Table 1. The median donor age was 53 years (IQR: 45–61 years), and 66.2% were female. Most donors were partners, parents, or siblings. Overall, 40.1% (*n* = 747) had at least 1 preexisting condition. Arterial hypertension treated with ≥ 1 antihypertensive agent was present in 15.5% (*n* = 288), and 2.4% (*n* = 45) had other cardiovascular diseases such as coronary artery disease or arrhythmia. Dyslipidemia occurred in 4.9%, and 7 donors had T2DM. Predonation obesity was observed in 11% (*n* = 205).

**Table 1:** Baseline characteristics (*N* = 1863)

Variable	Overall
Age at donation (yr)	53 (45–61)
Age grouping at donation, *n* (%)	
< 40 yr	242 (13.0)
40–50 yr	566 (30.4)
51–60, yr	605 (32.5)
61–70, yr	385 (20.6)
> 70 yr	65 (3.5)
Sex, *n* (%)	
Female	1233 (66.2)
Male	630 (33.8)
Donor-recipient relation, *n* (%)	
Parents	469 (25.2)
Siblings	410 (22.0)
Other relatives	75 (4.0)
Partners	690 (37.0)
Other non-relatives	219 (11.8)
KPD, *n* (%)	36 (1.9)
Altruistic donors, *n* (%)	10 (0.54)
Era of donation	
1993–2000, *n* (%)	341 (18.3)
2001–2009, *n* (%)	718 (38.5)
2010–2018, *n* (%)	804 (43.2)
Pre-existing diseases, *n* (%)^a^	747 (40.1)
Hypertension, *n* (%)	288 (15.5)
Cardio-vascular diseases, *n* (%)	45 (2.4)
Dyslipidemia, *n* (%)	92 (4.9)
T2DM, *n* (%)	7 (0.4)
Obesity, *n* (%)^b^	205 (11.0)
Other	375 (20.1)
Any medication taken, yes, *n* (%)	670 (36.0)
Smoking, yes, *n* (%)^c^	124 (14.8)
Kidney function before donation	
SCr (μmol/l)	67 (58–77)
eGFR, CKD-EPI (ml/min per 1.73 m^2^)	96 (85–105)
eGFR category, CKD-EPI (ml/min per 1.73m^2^)^d^	
≥ 90, *n* (%)	1222 (66.5)
80–89, *n* (%)	317 (17.3)
70–79, *n* (%)	188 (10.2)
60–69, *n* (%)	92 (5.0)
50–59, *n* (%)	18 (1.0)
UPCR (mg/mmol)^e^	7.7 (5.9–10.8)
UAC (mg/mmol)^f^	0.6 (0.4–1.0)
Proteinuria category^g^	
None/mild, *n* (%)	1236 (88.3)
Moderate, *n* (%)	163 (11.6)
Severe, *n* (%)	1 (0.1)
Systolic blood pressure (mm Hg)	126 (118–135)
Diastolic blood pressure (mm Hg)	78 (71–83)
Weight (kg)	70 (62–80)
BMI (kg/m^2^)^h^	25.0 (22.7–27.8)
BMI < 25 kg/m^2^, *n* (%)	917 (49.5)
BMI 25–29.9 kg/m^2^, *n* (%)	732 (39.4)
BMI 30–34.9 kg/m^2^, *n* (%)	181 (9.8)
BMI ≥ 35 kg/m^2^, *n* (%)	24 (1.3)

CKD-EPI, Chronic Kidney Disease Epidemiology Collaboration; eGFR, estimated glomerular filtration rate (calculated by the CKD-EPI 2009 formula; in ml/min per 1.73m^2^ of body surface); KPD, kidney paired donation; SCr, serum creatinine.

Values are median (IQR) unless otherwise stated.

a In total, 747 (40.1%) living kidney donors had one or more preexisting diseases.

b Obesity was defined as BMI ≥30 kg/m^2^ according to WHO criteria.

c Smoking behavior is known of n = 838 (45.0%) living kidney donors.

d eGFR was missing for n = 26 donors.

e UPCR, urine protein-to-creatinine ratio.

f UACR, urine albumin-to-creatinine ratio, which was only calculated for undiluted urine samples (i.e., urine creatinine ≥ 3 mmol/l; n = 1400).

g Proteinuria categories were defined by UPCR and/or UACR based on the Kidney Disease Improving Global Outcomes (KDIGO) definitions as none/mild (UPCR < 15 mg/mmol, and/or UACR < 3 mg/mmol), moderate (UPCR 15–50 mg/mmol, and/or UACR 3–30 mg/mmol), and severe (UPCR > 50 mg/mmol, and/or UACR > 30 mg/mmol).

h BMI = Body mass index (in total n = 1854 values available).

Median serum creatinine was 67 μmol/l (IQR: 58–77 μmol/l), and median eGFR was 96 ml/min per 1.73 m^2^ (IQR: 85–105 ml/min per 1.73 m^2^); 66.5% had eGFR ≥ 90 ml/min per 1.73 m^2^ and 97.6% had eGFR ≥ 60 ml/min per 1.73 m^2^, whereas only 1% (n = 18) had eGFR < 60 ml/min per 1.73 m^2^. Most donors had none or mild proteinuria. Median systolic and diastolic blood pressures were 126 mm Hg (IQR: 118–135 mm Hg) and 78 mm Hg (IQR: 71–83 mm Hg), respectively. Median BMI was 25.0 kg/m^2^ (IQR: 22.7–27.8 kg/m^2^), with 9.8% having a BMI between 30–34.9 kg/m^2^ and 1.3% a BMI ≥ 35 kg/m^2^.

### Longitudinal Follow-Up Assessments

Median follow-up of the entire cohort was 12 years (IQR: 7.0–18.0 years). Median values (IQR) of kidney function, blood pressure, weight, and BMI across scheduled follow-up visits are shown in Table 2. The number of donors with available data decreased over time, reflecting routine clinical follow-up patterns, and leading to varying sample composition across time points, which may also reflect temporal cohort effects. In total, 13,222 follow-up visits were recorded between 1 and 30 years postdonation. The 1863 donors contributed 9482 serum creatinine measurements after the index date during follow-up.

**Table 2:** Median values in living kidney donors according to the follow-up visits over time (*N* = 13,222)

Variable	Before donation (*n* = 1863)	1 yr (*n* = 1714)	3 yr (*n* = 1666)	5 yr (*n* = 1674)	7 yr (*n* = 1456)	10 yr (*n* = 1144)	12 yr (*n* = 964)	14 yr (781)
Age (yr)	53 (45–61)	55 (47–62)	56 (49–64)	59 (51–66)	60 (53–68)	63 (55–70)	64 (56–71)	65 (58–72)
Sex, *n* (%)								
Female	1233 (66.2)	1134 (66.2)	1108 (66.5)	1110 (66.3)	983 (67.5)	773 (67.6)	653 (67.7)	533 (68.2)
Male	630 (33.8)	580 (33.8)	558 (33.5)	564 (33.7)	473 (32.5)	371 (32.4)	311 (32.3)	248 (31.8)
Kidney function								
SCr (μmol/l)	67 (58–77)	98 (86–112)	94 (84–109)	92 (81–105)	89 (79–103)	88 (77–102)	87 (76–100)	85 (76–99)
eGFR (ml/min per 1.73m^2^)	96 (85–105)	62 (54–71)	63 (55–73)	65 (56–75)	66 (57–75)	66 (57–76)	67 (57–76)	66 (57–77)
eGFR category (ml/min per 1.73 m^2^)								
≥ 90, *n* (%)	1222 (66.5)	86 (5.1)	101 (6.2)	109 (6.6)	111 (7.8)	82 (7.3)	78 (8.3)	65 (8.4)
60–89, *n* (%)	597 (32.5)	854 (50.9)	877 (53.4)	937 (56.9)	844 (58.9)	690 (61.7)	561 (59.5)	456 (59.1)
30–59, *n* (%)	18 (1.0)	739 (44.0)	662 (40.3)	600 (36.4)	475 (33.1)	344 (30.8)	301 (31.9)	244 (31.7)
< 30, *n* (%)	0 (0)	0 (0)	2 (0.1)	1 (0.1)	3 (0.2)	2 (0.2)	3 (0.3)	6 (0.8)
UPCR (mg/mmol)	7.7. (5.9–10.8)	9.8 (7.0–13.9)	9.4 (6.9–13.2)	9.9 (7.1–13.7)	9.8 (7.2–13.6)	10.0 (7.3–13.8)	10.1 (7.5–14.0)	10.3 (7.6–14.1)
UACR (mg/mmol)	0.6 (0.4–1.0)	0.6 (0.4–1.2)	0.6 (0.3–1.2)	0.6 (0.4–1.2)	0.6 (0.4–1.3)	0.7 (0.4–1.5)	0.8 (0.4–1.5)	0.8 (0.5–1.6)
Proteinuria category^a^								
None/mild	1268 (90.6)	1048 (79.6)	1088 (82.1)	1094 (80.6)	931 (79.8)	742 (80.0)	610 (78.0)	504 (77.4)
Moderate	131 (9.3)	261 (19.8)	226 (17.1)	249 (18.4)	219 (18.8)	177 (19.1)	156 (19.9)	131 (20.1)
Severe	1 (0.1)	8 (0.6)	11 (0.8)	14 (1.0)	16 (1.4)	8 (0.9)	16 (2.1)	16 (2.5)
Albuminuria category^b^								
None/mild	1333 (95.4)	1217 (92.3)	1220 (92.0)	1232 (90.6)	1048 (89.9)	816 (87.7)	689 (88.0)	559 (85.8)
Moderate	63 (4.5)	100 (7.5)	103 (7.8)	117 (8.6)	109 (9.3)	111 (12.0)	85 (10.9)	83 (12.7)
Severe	1 (0.1)	2 (0.2)	3 (0.2)	11 (0.8)	9 (0.8)	3 (0.3)	9 (1.1)	10 (1.5)
Blood pressure (mm Hg)								
Systolic	126 (118–135)	128 (118–137)	128 (120–138)	129 (120–139)	130 (120–140)	130 (121–140)	130 (121–140)	13 (121–140)
Diastolic	78 (71–83)	80 (75–86)	80 (75–87)	80 (75–86)	80 (76–87)	80 (75–87)	80 (75–86)	80 (75–86)
Weight (kg)	70 (62–80)	71 (62–81)	71 (62–82)	72 (63–81)	72 (63–82)	71 (62–82)	73 (62–83)	73 (61–83)
BMI (kg/m^2^)	25 (22.7–27.8)	25.3 (22.7–28.1)	25.4 (22.8–28.5)	25.5 (22.9–28.4)	25.8 (23.0–28.8)	25.5 (22.9–28.8)	25.9 (22.9–29.0)	26.0 (22.9–29.2)
BMI categories								
< 25 kg/m^2^	917 (49.5)	723 (47.1)	678 (46.7)	646 (43.5)	537 (42.2)	448 (44.0)	355 (41.5)	287 (40.6)
25–29.9 kg/m^2^	732 (39.4)	590 (38.5)	535 (36.9)	592 (39.9)	510 (40.0)	382 (37.5)	333 (38.9)	273 (38.7)
30–34.9 kg/m^2^	181 (9.8)	191 (12.4)	202 (13.9)	204 (13.8)	193 (15.1)	154 (15.1)	139 (16.3)	110 (15.6)
≥ 35 kg/m^2^	24 (1.3)	30 (2.0)	37 (2.5)	41 (2.8)	34 (2.7)	35 (3.4)	28 (3.3)	36 (5.1)
Variable	16 yr (*n* = 617)	18 yr (*n* = 470)	20 yr (*n* = 351)	22 yr (*n* = 239)	24 yr (*n* = 145)	26 yr (*n* = 84)	28 yr (*n* = 42)	30 yr (*n* = 12)
Age (yr)	67 (60–74)	68 (61–75)	69 (63–77)	71 (64–78)	71 (64–80)	72 (66–82)	74 (67–83)	76 (66–83)
Sex, *n* (%)								
Female	422 (68.4)	319 (67.9)	237 (67.5)	166 (69.5)	98 (67,6)	59 (70.2)	30 (71.4)	9 (75.0)
Male	195 (31.6)	151 (32.1)	114 (32.5)	73 (30.5)	47 (32.4)	25 (29.8)	12 (28.6)	3 (25.0)
Kidney function								
SCr (μmol/l)	86 (75–99)	85 (76–99)	85 (75–98)	84 (74–95)	82 (73–95)	81 (73–96)	84 (72–101)	89 (73–102)
eGFR (ml/min per 1.73 m^2^)	66 (56–77)	65 (55–77)	65 (55–77)	67 (57–76)	67 (58–78)	66 (56–79)	69 (52–79)	69 (46–74)
eGFR category (ml/min per 1.73 m^2^)								
≥ 90, *n* (%)	50 (8.2)	36 (7.8)	23 (6.7)	13 (5.5)	16 (11.4)	8 (9.5)	3 (7.3)	1 (8.3)
60–89, *n* (%)	356 (58.6)	262 (56.6)	190 (55.1)	141 (59.8)	87 (61.7)	49 (58.3)	24 (58.6)	6 (50.0)
30–59, *n* (%)	197 (32.4)	157 (33.9)	123 (35.6)	78 (33.0)	35 (24.8)	24 (28.6)	13 (31.7)	5 (41.7)
< 30, *n* (%)	5 (0.8)	8 (1.7)	9 (2.6)	4 (1.7)	3 (2.1)	3 (3.6)	1 (2.4)	0 (0)
UPCR (mg/mmol)	10.4 (7.8–15.1)	11.1 (7.9–16.1)	10.9 (8.6–15.8)	11.7 (8.0–15.2)	11.4 (8.0–17.8)	11.3 (8.6–19.3)	11.3 (7.3–17.3)	12.9 (9.7–17.6)
UACR (mg/mmol)	0.9 (0.5–2.0)	0.9 (0.5–1.9)	1.0 (0.5–2.2)	1.0 (0.6–2.2)	0.9 (0.5–3.3)	1.3 (0.5–3.3)	1.1 (0.5–2.5)	0.6 (0.3–1.8)
Proteinuria category^a^								
None/mild	381 (75.0)	278 (71.4)	188 (70.4)	147 (74.2)	74 (66.1)	45 (64.3)	20 (58.8)	7 (70.0)
Moderate	115 (22.6)	96 (24.7.)	64 (24.0)	42 (21.2)	35 (31.2)	23 (32.8)	12 (35.3)	2 (20.0)
Severe	12 (2.4)	15 (3.9)	15 (5.6)	9 (4.6)	3 (2.7)	2 (2.9)	2 (5.9)	1 (10.0)
Albuminuria category^b^								
None/mild	419 (82.1)	320 (82.5)	219 (82.0)	159 (80.3)	85 (74.6)	48 (67.6)	27 (79.4)	10 (100)
Moderate	82 (16.1)	58 (14.9)	36 (13.5)	33 (16.7)	29 (25.4)	21 (29.6)	7 (20.6)	0 (0)
Severe	9 (1.8)	10 (2.6)	12 (4.5)	6 (3.0)	0 (0)	2 (2.8)	0 (0)	0 (0)
Blood pressure (mm Hg)								
Systolic	131 (123–140)	133 (125–142)	133 (124–142)	134 (122–142)	132 (121–142)	135 (125–150)	130 (121–140)	134 (126–144)
Diastolic	80 (73–86)	80 (75–86)	80 (74–86)	80 (73–85)	80 (72–87)	80 (76–89)	76 (70–85)	82 (75–87)
Weight (kg)	72 (62–83)	73 (63–83)	73 (63–85)	73 (62–83)	74 (65–84)	77 (63–87)	75 (57–87)	71 (58–84)
BMI (kg/m^2^)	26.0 (23.0–29.3)	26.2 (23.0–29.3)	26.6 (23.4–29.8)	26.5 (22.8–29.8)	26.7 (24.1–30.0)	28.1 (24.0–31.1)	26.5 (23.4–30.3)	26.2 (22.8–30.5)
BMI categories								
< 25 kg/m^2^	220 (39.6)	166 (39.2)	112 (35.3)	82 (38.9)	42 (33.1)	24 (32.4)	12 (31.6)	5 (45.4)
25–29.9 kg/m^2^	212 (38.1)	169 (40)	128 (40.4)	79 (37.4)	52 (40.9)	28 (37.9)	16 (42.1)	3 (27.3)
30–34.9 kg/m^2^	101 (18.2)	70 (16.5)	57 (18.0)	37 (17.5)	28 (22.1)	18 (24.3)	8 (21.0)	2 (18.2)
≥ 35 kg/m^2^	23 (4.1)	18 (4.3)	20 (6.3)	13 (6.2)	5 (3.9)	4 (5.4)	2 (5.3)	1 (9.1)

BMI, body mass index; CKD-EPI, Chronic Kidney Disease Epidemiology Collaboration; eGFR, estimated glomerular filtration rate (calculated by the CKD-EPI 2009 formula in ml/min per 1.73m^2^ of body surface); SCr, serum creatinine; UPCR, urine protein-to-creatinine ratio; UACR, urine albumin-to-creatinine ratio (which were only calculated for donors with undiluted urine, i.e., urine creatinine ≥ 3 mmol/l).

Values are median (IQR) unless otherwise stated.

Values represent cross-sectional summaries at each time point and are based on donors with available data at the respective follow-up.

a Proteinuria categories were defined by UPCR based on the Kidney Disease Improving Global Outcomes (KDIGO) definitions as none/mild (UPCR < 15 mg/mmol), moderate (UPCR 15-50 mg/mmol), and severe (UPCR > 50 mg/mmol).

b Albuminuria categories were defined based on the KDIGO definitions as none/mild (UACR < 3 mg/mmol), moderate (UACR 3–30 mg/mmol), and severe (UACR > 30 mg/mmol).

### Evolution of Kidney Function After Donation

**Figure 2:**
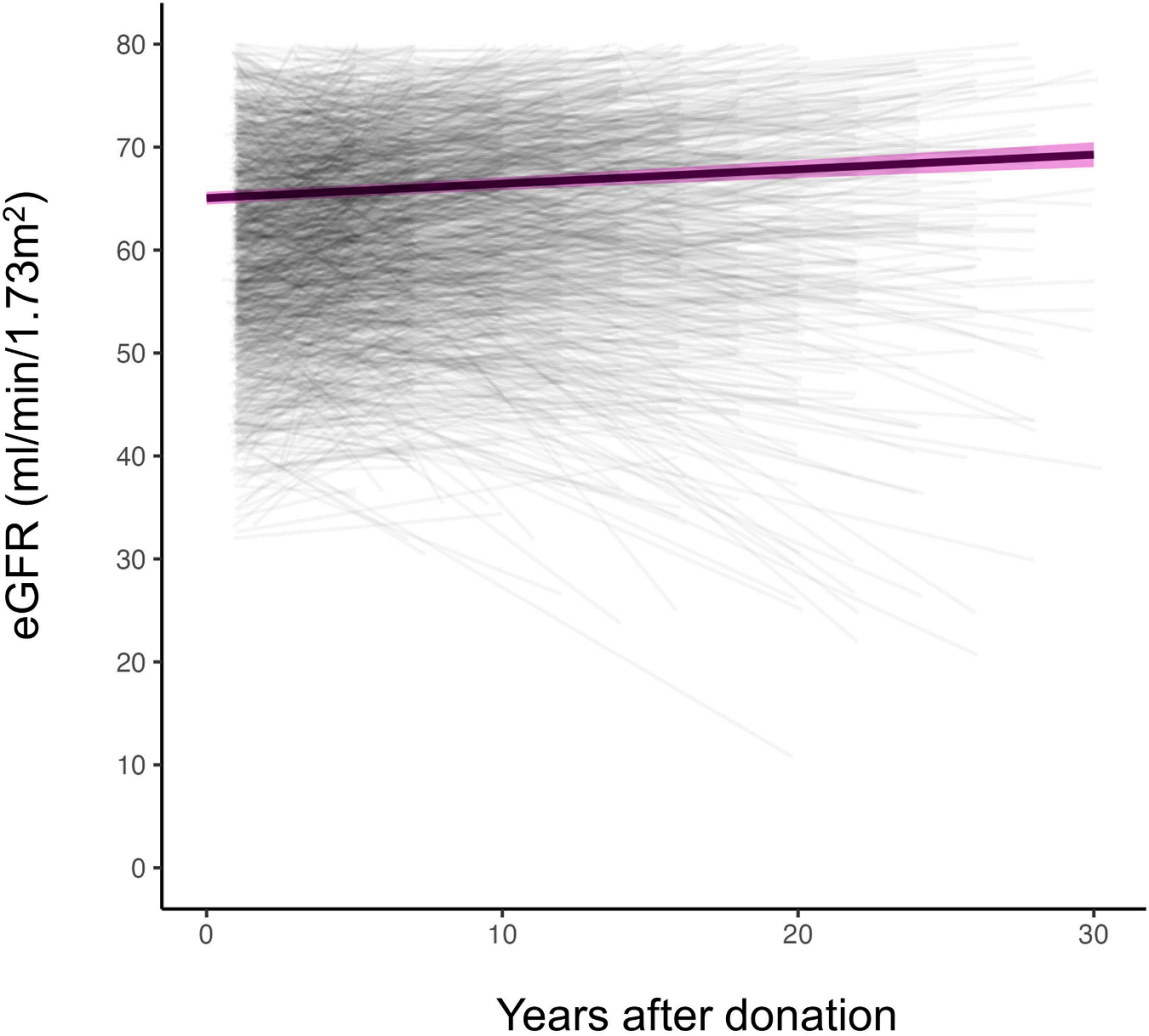
Population-based trajectory of eGFR after donation. The solid line represents the model-estimated mean eGFR over time derived from a linear mixed-effects model, and the shaded area indicates 95% confidence interval. Analyses include measurements from 1 year postdonation onward. eGFR, estimated glomerular filtration rate.

**Figure 3a-c:**
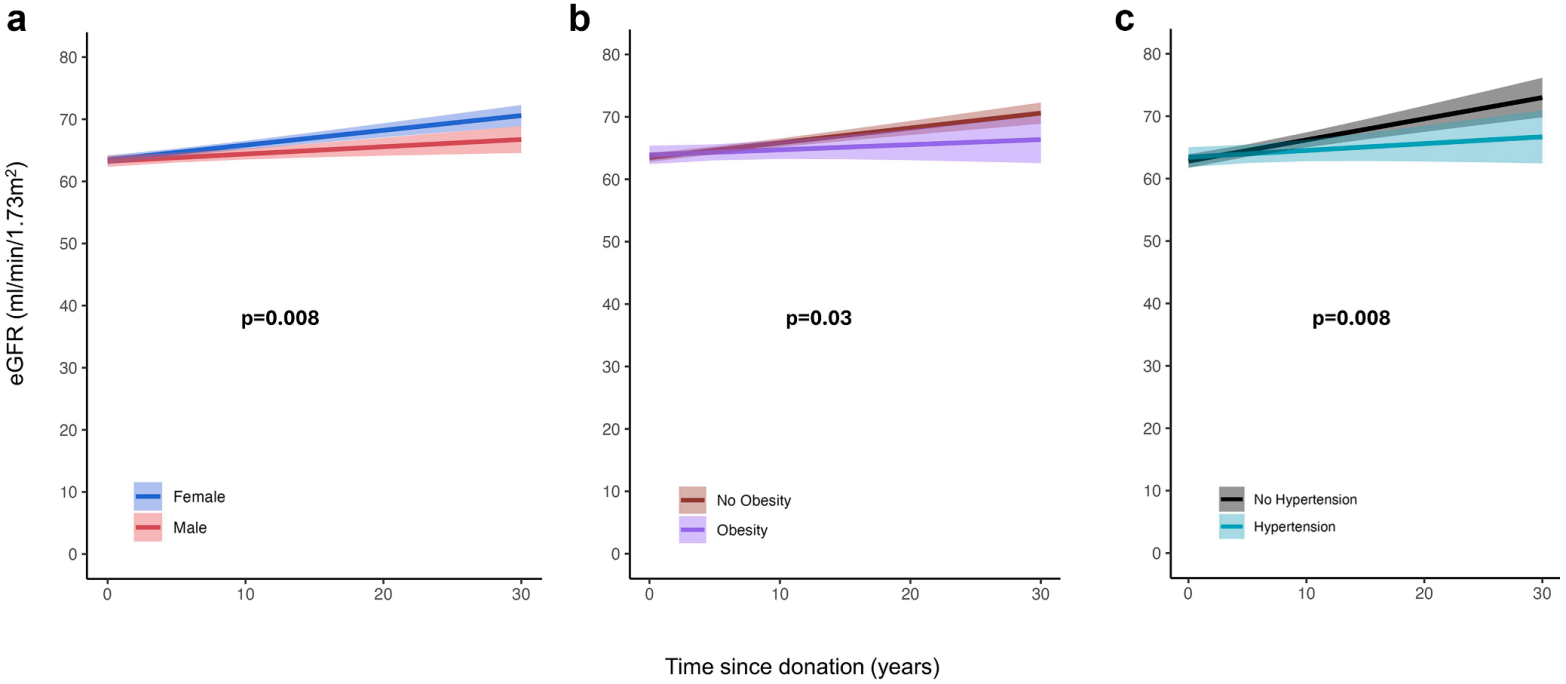
Estimated trajectories of eGFR after LKD, stratified by (a) donor sex, (b) obesity (BMI ≥ 30 kg/m^2^), and (c) predonation hypertension. Lines represent model-estimated mean eGFR trajectories from linear mixed-effects models, and shaded areas indicate 95% confidence intervals. Analyses include measurements from 1 year postdonation onward. *P*-values refer to differences in the rate of eGFR change over time between groups. eGFR, estimated glomerular filtration rate; LKD, living kidney donation.

The population-based slope of eGFR after donation is shown in Figure 2, and median serum creatinine and eGFR values at follow-up timepoints are summarized in Table 2. Over follow-up, eGFR remained stable. The average annual change in eGFR was +0.14 ml/min per 1.73 m^2^ (95% CI: 0.10–0.18 ml/min per 1.73 m^2^; Figure 2). However, eGFR slopes differed by donor characteristics with small but statistically significant differences observed between female and male donors, presence of obesity and patients without hypertension (Figure 3a–c). In detail, female donors had a higher average eGFR increase than males (+0.22 vs. +0.10 ml/min per 1.73 m^2^ per year; *P* = 0.008; Figure 3a, Supplementary Table S1). Donors without obesity showed a greater increase than obese donors (+0.20 vs. +0.04 ml/min per 1.73 m^2^ per year; *P* = 0.03; Figure 3b, Supplementary Table S1), and hypertensive donors had a smaller increase than normotensive donors (+0.06 vs. +0.29 ml/min per 1.73 m^2^ per year; *P* = 0.008; Figure 3c, Supplementary Table S2). Differences were not significant for age (*P* = 0.05), whereas slopes varied across baseline eGFR values, with some pairwise comparisons reaching statistical significance (Supplementary Table S1).

Based on the last available SCr, 17 donors (0.9%) had an eGFR < 30 ml/min per 1.73 m^2^; 3 of them required renal replacement therapy. Most donors (56.3%) had an eGFR between 60–89 or 30–59 ml/min per 1.73 m^2^ (35.9%; Supplementary Table S3). Donors who reached an eGFR < 30 ml/min per /1.73 m^2^ were older at donation (62 vs. 53 years; *P* < 0.0001), had lower predonation eGFR (83 vs. 96 ml/min per 1.73 m^2^; *P* = 0.0004), were more often male, predonation hypertensive (41.2% vs. 15.2%; *P* = 0.01), and had higher BMI at the time of donation (Table 3). After donation, they had more severe proteinuria (29.4% vs. 1.3%; *P* < 0.0001), new-onset cardiovascular disease (41.2% vs. 7.1%; *P* = 0.0001), and new-onset T2DM (25.5% vs. 5.9%; *P* = 0.02), whereas new-onset hypertension did not differ (*P* = 0.15; Table 3).

**Table 3:** Characteristics of donors stratified by reaching a low eGFR < 30 ml/min per 1.73m2 at last follow-up vs. ≥ 30 ml/min per 1.73 m^2^(*N* = 1863)

Variable	Donors with low eGFR < 30 ml/min per 1.73 m^2^ (*n* = 17)	Donors with eGFR ≥ 30 ml/min per 1.73 m^2^ (*n* = 1846)	*P*-value
Age at donation (yr)	62 (61–63)	53 (45–61)	0.0001
Age at last follow-up (yr)	81 (79–84)	66 (58–74)	< 0.0001
Sex, *n* (%)			
Male	12 (70.6)	618 (33.5)	0.003
Female	5 (29.4)	1228 (66.5)	
Pre-existing hypertension (yes), *n* (%)	7 (41.2)	281 (15.2)	0.01
New-onset hypertension (yes), *n* (%)^a^	7 (41.2)	444 (24.1)	0.15
New-onset cardio-vascular diseases (yes), *n* (%))	7 (41.2)	131 (7.1)	0.0001
New onset T2DM (yes), *n* (%)	4 (23.5)	109 (5.9)	0.02
New onset malignancies (yes), *n* (%)	3 (17.7)	141 (7.6)	0.14
Kidney function parameters			
SCr before donation (μmol/l)	83 (78–95)	66 (58–77)	< 0.0001
predonation eGFR (ml/min per 1.73 m^2^)	83 (67–93)	96 (86–105)	0.0004
SCr last follow-up (μmol/l)	202 (170–241)	88 (77–103)	< 0.0001
eGFR (ml/min per 1.73 m^2^) last follow-up	24 (19–28)	65 (55–75)	< 0.0001
Persistent severe proteinuria (yes), *n* (%)^b^	5 (29.4)	23 (1.3)	< 0.0001
Body weight before donation (kg)	77 (72–90)	70 (62–80)	0.004
Predonation BMI (kg/m^2^)	27.8 (25.4–30.7)	25.0 (22.7–27.7)	0.002

BMI, body mass index; eGFR, estimated glomerular filtration rate (calculated by the CKD-EPI formula; in ml/min per 1.73 m^2^ of body surface); CKD-EPI, Chronic Kidney Disease Epidemiology Collaboration; SCr, serum creatinine.

Values are median (IQR) unless otherwise stated.

a For new-onset hypertension, only treated donors from 1 year onward were included.

b Persistent severe proteinuria was defined as urine protein-to-creatinine ratio >50mg/mmol and/or urine albumin-to-creatinine ratio >30mg/mmol according to the Kidney Disease Improving Global Outcomes (KDIGO) definitions, which were only calculated for donors with undiluted urine (i.e., urine creatinine ≥3 mmol/l).

### Occurrence of New-Onset Arterial Hypertension and Proteinuria Postdonation

New-onset hypertension occurred in 548 (29.4%) living kidney donors during a median follow-up of 6.8 years (IQR: 3.0–11.9 years), and 521 (28.0%) received antihypertensive therapy. To minimize misclassification, cases identified within the first year postdonation were excluded. After this exclusion, 475 (25.5%) donors developed hypertension during a median follow-up of 7.1 years (IQR: 5.0–12.1 years), and 451 (24.2%) required treatment.

Differences among living kidney donors stratified by treated new-onset hypertension are summarized in Table 4. The incidence was higher in men than in women (39.9% vs. 31.9%; *P* = 0.002). At donation, donors who later developed hypertension were older (55 vs. 52 years; *P* < 0.0001), and more likely to have donated in earlier eras between 1993-2000 (*P* < 0.0001). They also had higher baseline body weight and BMI (25.6 vs. 24.8 kg/m^2^; *P* = 0.003), whereas obesity and dyslipidemia prevalence did not differ significantly. At last follow-up, hypertensive donors had lower eGFR (61 vs. 66 ml/min per 1.73 m^2^; *P* < 0.0001), higher UPCR and UACR (both *P* < 0.0001), and more frequent persistent severe proteinuria (3.3% vs. 0.9%; *P* = 0.001). Median observation time was longer among hypertensive donors (16.1 vs. 11.9 years; *P* < 0.0001), whereas the median time to onset (7.1 years; IQR: 5.0–12.1 years) suggests that most cases arose within the overall follow-up period. Therefore, differences in hypertension incidence are unlikely to be solely attributable to longer observation, although longer follow-up may have contributed to variations in renal outcomes at last assessment.

**Table 4:** Characteristics of donors stratified by reaching new-onset arterial hypertension versus all other living kidney donors(*N* = 1863)

Variable	New-onset arterial hypertension “yes” (*n* = 451)	New-onset arterial hypertension “no” (*n* = 1412)	*P*-value
Age at donation (yr)	55 (48–61)	52 (45–61)	< 0.0001
Sex, *n* (%)			
Male	180 (39.9)	450 (31.9)	0.002
Era of donation			
1993–2000, *n* (%)	142 (31.5)	199 (14.1)	< 0.0001
2001–2009, *n* (%)	197 (43.7)	521 (36.9)	
2010–2018, *n* (%)	112 (24.8)	692 (49.0)	
Predonation body weight (kg)	72 (64–81)	70 (61–80)	0.009
Predonation BMI (kg/m^2^)	25.6 (23.0–28.1)	24.8 (22.5–27.7)	0.003
Obesity before donation (yes), *n* (%)^a^	51 (11.3)	154 (10.9)	0.32
Dyslipidemia before donation (yes), *n* (%)	16 (3.6)	76 (5.4)	0.13
Blood pressure values last follow-up (mm Hg)			
Systolic	135 (125–143)	130 (120–140)	< 0.0001
Diastolic	81 (75–87)	80 (75–87)	0.47
Kidney function parameters last follow-up			
SCr (μmol/l)	91 (78–108)	87 (77–102)	0.004
eGFR (ml/min per 1.73 m^2^)	61 (51–72)	66 (56–76)	< 0.0001
UPCR (mg/mmol)	12.6 (9.0–18.3)	10.8 (7.6–15.3)	< 0.0001
UACR (mg/mmol)	0.9 (0.4–2.3)	0.6 (0.3–1.3)	< 0.0001
Persistent severe proteinuria (yes), *n* (%)^b^	15 (3.3)	13 (0.9)	0.001
eGFR last follow-up < 30 ml/min per 1.73 m^2^ (yes), *n* (%)	7 (1.6)	10 (0.7)	0.15
Entire follow-up (yr)	16.1 (11.9–21.9)	11.9 (7.0–16.0)	< 0.0001

BMI, body mass index; eGFR, estimated glomerular filtration rate (calculated by the CKD-EPI formula; in ml/min per 1.73m^2^ of body surface); SCr, serum creatinine; UPCR, urine protein-to-creatinine ratio; UACR, urine albumin-to-creatinine ratio.

Values are median (IQR) unless otherwise stated.

a Obesity was defined as BMI ≥30 kg/m^2^ according to WHO criteria.

b Persistent severe proteinuria was defined as urine protein-to-creatinine ratio > 50 mg/mmol and/or urine albumin-to-creatinine ratio > 30 mg/mmol according to the Kidney Disease Improving Global Outcomes (KDIGO) definitions. UPCR, UACR as well as persistent proteinuria were only calculated for undiluted urine samples (i.e., urine creatinine ≥ 3 mmol/l).

### Endpoint-Free Donor Survival

Donor baseline characteristics stratified by reaching the combined postdonation end point are presented in Supplementary Table S4. During the observation, no early deaths occurred, but 3 donors (0.2%) developed KF requiring renal replacement therapy, 20.6–24.1 years after donation. Overall, 17 donors (0.9%) had eGFR < 30 ml/min per 1.73 m^2^, and 28 (1.5%) showed persistent severe proteinuria (Table 3). In total, 40 donors (2.1%) reached the combined end point. These donors were older at last observation (81 vs. 66 years; *P* < 0.0001) and had longer follow-up (19.0 vs. 12.0 years; *P* < 0.0001) (Table 5). They more frequently developed new-onset hypertension (55.0% vs. 28.9%; *P* = 0.0007), T2DM (22.5% vs. 5.7%; *P* = 0.0004), cardiovascular disease (35.0% vs. 6.8%; *P* < 0.0001), and malignancy (22.5% vs. 7.4%; *P* = 0.003). At last follow-up, they had lower eGFR (median 35 vs. 65 ml/min per 1.73 m^2^; *P* < 0.0001) and higher proteinuria and albuminuria (UPCR 80.2 vs. 11.1 mg/mmol; UACR 33.2 vs. 0.6 mg/mmol; both *P* < 0.0001), with 42.5% showing eGFR < 30 ml/min per 1.73 m^2^ compared with none in the reference group (*P* < 0.0001).

**Table 5:** Outcomes of donors stratified by reaching the combined end point postdonation(*N* = 1863)

Variable	Donors reaching the combined end point postdonation (*n* = 40)	Donors with uneventful postdonation course (*n* = 1823)	*P*-value
Time form donation (yr)	19.0 (14.1–22.0)	12.0 (7.0–17.9)	< 0.0001
Age at last follow-up (yr)	81 (72–84)	66 (58–74)	< 0.0001
New-onset hypertension (yes), *n* (%)	22 (55.0)	526 (28.9)	0.0007
New-onset T2DM (yes), *n* (%)	9 (22.5)	104 (5.7)	0.0004
New-onset cardio-vascular diseases (yes), *n* (%)	14 (35.0)	124 (6.8)	< 0.0001
New-onset malignancies (yes), *n* (%)	9 (22.5)	135 (7.4)	0.003
Kidney function last follow-up			
SCr (μmol/l)	147 (108–190)	87 (77–102)	< 0.0001
eGFR, CKD-EPI (ml/min per 1.73 m^2^)	35 (24–53)	65 (55v76)	< 0.0001
eGFR categories (ml/min per 1.73 m^2^)			
≥ 90, *n* (%)	0 (0)	129 (7.1)	< 0.0001
60–89, *n* (%)	6 (15.0)	1042 (57.1)	
30–59, *n* (%)	17 (42.5)	652 (35.8)	
< 30, *n* (%)	17 (42.5)	0 (0)	
UPCR (mg/mmol)^a^	80.2 (51.5–118.6)	11.1 (7.9–15.7)	< 0.0001
UACR (mg/mmol)^b^	33.2 (10.3–71.8)	0.6 (0.4–1.4)	< 0.0001
Blood pressure last follow-up (mm Hg)			
Systolic blood pressure	137 (121–145)	131 (121–141)	0.36
Diastolic blood pressure	78 (70–82)	80 (75–87)	0.005
Body weight last follow-up (kg)	78 (69–86)	72 (62–82)	0.01
BMI last follow-up (kg/m^2^)	27.1 (25.0–30.1)	25.8 (23.0–29.0)	0.07

BMI, body mass index; eGFR, estimated glomerular filtration rate (calculated by the CKD-EPI formula; in ml/min per 1.73m^2^ of body surface); CKD-EPI, Chronic Kidney Disease Epidemiology Collaboration; SCr, serum creatinine.

Values are median (IQR) unless otherwise stated.

a UPCR, urine protein-to-creatinine ratio and.

b UACR, urine albumin-to-creatinine ratio, which were only calculated for donors with undiluted urine (i.e., urine creatinine ≥3 mmol/l).

**Figure 4a-d:**
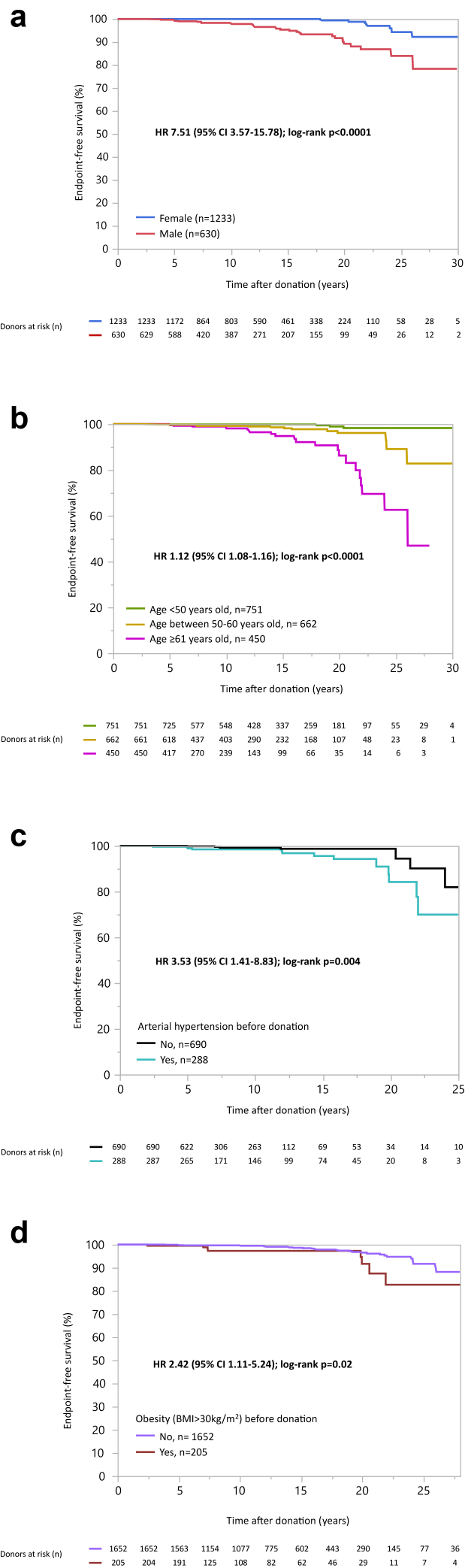
Endpoint-free donor survival of groups stratified by sex (4a), age grouping (4b), presence of predonation arterial hypertension yes vs. no (4c) as well as predonation obesity (BMI ≥ 30 kg/m^2^) yes vs. no (4d). Each additional year of age was associated with an 11% increase in risk (4b). CI, confidence interval; HR, hazard ratio.

Kaplan–Meier analysis showed significantly lower endpoint-free survival in male compared with female donors (*P* < 0.0001; Figure 4a) and across increasing age groups (*P* < 0.0001; Figure 4b). Donors with predonation hypertension also had lower end pointfree survival than those without baseline hypertension (*P* = 0.004; Figure 4c). Donors with unclear hypertension status were initially excluded to reduce bias; survival curves for hypertensive and normotensive donors were similar during the first decade but diverged thereafter, with hypertensive donors showing lower long-term endpoint-free survival probabilities. The number at risk declined over time in both groups, limiting precision in later intervals. When donors with unclear medical history were reclassified as normotensive (based on the absence of antihypertensive therapy), results remained consistent, showing significantly lower endpoint-free survival among hypertensive donors (*P* < 0.0001; Supplementary Figure S1). Differences between obese and nonobese donors were modest early after donation but became more apparent with extended follow-up (*P* = 0.02; Figure 4d). Interpretation was limited by the small number of obese donors remaining under observation beyond 10 years.

### Independent Predictors of Unfavorable Outcomes After Donation

In multivariable Cox proportional hazards analysis, male sex, older age, and predonation obesity were independently associated with an increased risk of the combined endpoint (Table 6). Male donors had a substantially higher hazard compared with females (hazard ratio [HR]: 7.25, 95% CI: 3.44–15.3; *P* < 0.0001). Each additional year of age was associated with an 11% increase in risk (HR: 1.11, 95% CI: 1.07–1.16; *P* < 0.0001), and predonation obesity conferred an approximately twofold higher risk (HR: 2.25, 95% CI: 1.02–4.92; *P* = 0.04).

**Table 6:** Independent predictors of the combined end point postdonation (*N* = 1863)

Variable	Donors with uneventful postdonation course (*n* = 1823)	Donors reaching the combined end point postdonation (*n* = 40)	Univariate Cox proportional analysis Hazard ratio (95% CI); *P*-value	Multivariable Cox proportional analysis^c^ Hazard ratio (95% CI); *P*-value
Predictors of combined end point				
Sex (male vs. female), *n* (%)	599 (32.9)/1224 (67.1)	31 (77.5)/9 (22.5)	7.51 (3.57–15.78); < 0.0001	7.25 (3.44–15.3); < 0.0001
Age (yr)				
Median (IQR)^a^	53 (45–61)	62 (56–65)	1.12 (1.08–1.16); < 0.0001	1.11 (1.07–1.16); < 0.0001
Era of donation, *n*				
2001–2009 vs. 1993–2000, *n*	704/322	14/19	1.26 (0.55–2.87); 0.59	1.40 (0.60–3.29); 0.44
2010–2018 vs. 1993–2000, *n*	797/322	7/19	3.10 (0.90–10.74); 0.07	2.28 (0.64–8.17); 0.20
2010–2018 vs. 2001–2009, *n*	797/704	7/14	2.47 (0.81–7.51); 0.11	1.63 (0.53–5.02); 0.40
Predonation obesity (BMI ≥ 30 kg/m^2^)^b^				
Yes vs. no, *n* (%)	197 (10.8)/1620 (89.2)	8 (20.0)/32 (80.0)	2.42 (1.11–5.24); 0.03	2.25 (1.02–4.92); 0.04

BMI, body mass index; CI, confidence interval.

a Per unit increase in the explanatory variable.

b Predonation BMI was available of *n* = 1857 donors.

c The last column represents the whole multivariable model (analyzed with *n* = 1857 donors).

In an extended multivariable model including predonation hypertension, obesity, and donation era (Supplementary Table S5), predonation arterial hypertension emerged as an independent predictor of the combined endpoint (HR: 3.42, 95% CI: 1.34–8.68; *P* = 0.01). In this model, predonation obesity showed an elevated but longer statistically significant association (HR: 2.02, 95% CI: 0.72–5.64; *P* = 0.18). However, after exclusion of donors with unclear hypertension history, only 22 donors experienced an unfavorable outcome, limiting the feasibility of including this variable in the main multivariable model.

## Discussion

In this large prospective national cohort study with up to 3 decades of longitudinal follow-up, we show that long-term kidney function after LKD remains excellent, with only 0.2% developing KF, and < 1% reaching an eGFR < 30 ml/min per 1.73 m^2^. These findings extend previous reports demonstrating that KF after donation is rare in carefully selected donors.^15^^,^^42^^,^^43^ Donor nephrectomy causes an immediate reduction in GFR that is partly offset by compensatory hyperfiltration, resulting in a sustained net reduction of approximately 25% to 40%.^34^^,^^44^, ^45^, ^46^, ^47^ This was recently confirmed in our own cohort 12 months after LKD.^14^ If transplant programs accept donors with GFR <80 ml/min per 1.73 m^2^, some—particularly older individuals with limited adaptive capacity—may later reach a GFR < 60 ml/min per 1.73 m^2^. Accordingly, KDIGO recommends individualized assessment for donor candidates with GFR 60-89 ml/min per 1.73 m^2^ based on their demographic and health profile relative to the program’s acceptable risk.^28^ A more relevant question than the acceptable GFR threshold is how kidney function evolves after donation over the longterm, whether its decline mirrors that seen in CKD or reflects normal aging, and which factors accelerate this loss. In our cohort, median eGFR largely remained within KDIGO GFR category G2, with a stable trajectory over time, underscoring fundamental differences between living donors and patients with CKD. Adaptive hyperfiltration appears sufficient to preserve renal reserve for decades, and the excellent health profile of accepted donors, together with the absence of ongoing renal insults, likely explains the lack of progressive decline despite some donors reaching eGFR < 60 ml/min per 1.73 m^2^. Thus, isolated postdonation reductions in GFR should not be interpreted as CKD. Importantly, KDIGO GFR categories were developed for populations with intrinsic kidney disease and are not directly applicable to otherwise healthy individuals who have undergone nephrectomy. In the absence of additional markers of kidney damage, such as proteinuria or progressive functional decline, reduced GFR after donation should not be equated with progressive CKD. Misinterpretation in this context may lead to unnecessary concern or inappropriate clinical classification.^47^^,^^48^

Our study captured long-term outcomes under a unified national framework, with > 13,000 follow-up visits. Kidney function remained stable over follow-up (average annual eGFR +0.14 ml/min per 1.73 m^2^; 95% CI: 0.10–0.18), consistent with previously published studies, even though the average age at donation in these studies was lower than in our cohort.^23^^,^^24^^,^^37^ Importantly, kidney function trajectories were not uniform but stratified by donor characteristics. Women and donors without predonation obesity or hypertension showed preserved or rising eGFR trajectories, whereas men and donors with hypertension or obesity had flatter slopes and lower attained eGFR later in follow-up. These patterns suggest that pre-existing vascular and metabolic stressors limit adaptive capacity and increase susceptibility of the remaining nephron mass to long-term hemodynamic burden.^49^, ^50^, ^51^ Accordingly, donor comorbidities rather than nephrectomy per se appear to drive long-term renal risk.

After exclusion of early cases, approximately onequarter of donors developed treated hypertension, more frequently among men and older donors. This is consistent with a large cohort study reporting a 24.2% incidence of hypertension over a mean follow-up of 16.6 years, with associations to proteinuria, lower eGFR, cardiovascular diseases, and mortality, but only rarely with KF.^52^ In our cohort, donors who developed hypertension had longer follow-up; however, the median onset of approximately 7 years lay within the main observation window of donors who remained normotensive, suggesting that surveillance bias alone may not fully explain this finding. Nevertheless, we cannot exclude that longer follow-up may have contributed to the higher observed prevalence of hypertension, as our data do not allow assessment of whether the incidence reaches a plateau over time. Overall, late-onset hypertension after donation appears relatively common, affects renal parameters, but seldom results in clinically significant renal endpoints when adequately treated. The question of causality, however, could not be addressed, as no appropriate control group was available to determine whether kidney donation itself represents an independent risk factor for hypertension. Previous studies with matched controls did not find differences, with the prevalence increasing in both groups over time.^23^^,^^37^^,^^53^ Sanchez *et al.*^52^ reported a significantly lower prevalence of postdonation hypertension than that expected in age-matched controls. Lentine *et al.*^54^ demonstrated a 30% to50% higher prevalence of hypertension in non-Hispanic black donors compared with non-Hispanic white donors, but no difference between non-Hispanic black donors and ethnically matched controls. Given the variability in population characteristics and donation practices across countries, adequately matched control groups remain essential for evaluating postdonation hypertension risk.

About 2% of donors reached the composite endpoint. These donors were older, had longer follow-up, and more frequently developed new-onset hypertension, T2DM, cardiovascular disease, and malignancy, suggesting that age-related comorbidity rather than donation alone explains the higher risk. Endpoint-free survival was lower in men, older donors, and those with predonation hypertension or obesity, with survival curves diverging only after a decade of follow-up. This lagged divergence likely reflects the gradual emergence of hypertensive nephrosclerosis and macrovascular disease, reinforcing that donor kidneys age under the same biological principles as native kidneys. A similar pattern was observed for predonation obesity. These findings are consistent with previous long-term observational studies showing that incident cardiovascular and metabolic conditions, rather than donation itself, account for most cases of late KF among donors.^55^

Consistent with observed patterns in endpoint-free survival, predonation hypertension emerged as a strong predictor in univariate analysis, although limited event numbers restricted its inclusion in the primary multivariable model. In that model, male sex, older age, and predonation obesity independently predicted the composite endpoint, whereas reinstating hypertension in a sensitivity analysis again showed a more than 3-fold increased risk as obesity lost significance. Together, these findings support predonation hypertension as an important determinant of late adverse renal outcomes. Predonation hypertension should be interpreted as a risk marker rather than a contraindication to donation in carefully selected individuals. Our findings suggest that while hypertensive donors may have an increased long-term risk of adverse renal outcomes, this risk appears to be modulated by baseline health status and comorbidity burden rather than nephrectomy alone. These data support a strategy of individualized donor selection and, importantly, more intensive long-term surveillance in donors with pre-existing hypertension rather than exclusion from donation per se.

This national cohort study provides a long-term perspective on LKD in Switzerland, characterized by a stringent national donor evaluation process and systematic postdonation follow-up. Strengths include unified multicenter surveillance, extended duration of follow-up spanning up to 3 decades, as well as the inclusion of donors with comorbidities representative of contemporary practice. Limitations include the absence of a healthy matched control group that underwent screening comparable to donor evaluation and, importantly, was not excluded based on potential contraindications to living donation. Ideally, such controls would also participate in equivalent long-term surveillance with regular follow-up visits; however, implementing this level of monitoring in healthy individuals is logistically challenging and rarely feasible in practice. In this context, the primary aim of this study was not to establish causal effects of donation per se, but to characterize long-term kidney function trajectories and clinical outcomes among living donors within a real-world national framework. Accordingly, our findings should be interpreted as complementary to, rather than a substitute for, studies employing matched nondonor control groups, which remain essential for causal inference. Beyond this aspect, our study population consisted mainly of Caucasian donors, reflecting the ethnicity of donors in our country. Other ethnic groups may differ from those of primarily white donors, as discussed by Lentine *et al.*^54^ Thus, we could not assess whether ethnicity influences kidney function trajectories and long-term risk. Nevertheless, our results were consistent with many large cohort studies conducted abroad, including mainly white donors.^15^^,^^42^^,^^43^^,^^52^ A further factor influencing generalizability relates to the healthcare context in which this cohort was embedded. Switzerland provides universal health coverage and mandates structured lifelong follow-up of living kidney donors, resulting in high adherence to regular monitoring and early detection of comorbidities. In healthcare systems without guaranteed long-term donor surveillance, outcomes may differ, particularly with respect to the timely diagnosis and management of hypertension, diabetes, or declining kidney function. The overall favorable outcomes observed in our cohort may therefore, in part, reflect the strength of this national follow-up infrastructure. These findings underscore that long-term donor safety is not solely determined by baseline donor selection but also by the availability of sustained postdonation care, which should be considered when extrapolating our results to other settings. Incomplete follow-up at later time points may introduce selection bias because of changing sample composition and should be considered when interpreting long-term estimates. Finally, precision declined in the longest follow-up intervals because of attrition in donors at risk, but effect sizes and directions were consistent with large international cohorts, supporting the robustness of our findings.

In conclusion, KF was rare despite the inclusion of donors with hypertension and obesity, suggesting that broader donor eligibility was not associated with a substantial increase in renal risk in this cohort. Long-term outcomes were primarily associated with donor characteristics rather than definitively attributable to nephrectomy itself, with adverse events clustering among older, male, hypertensive, and obese individuals. These findings support the ethical and clinical justification for living donation and underscore the importance of individualized counseling and standardized long-term follow-up within national living donor programs to ensure optimal donor safety and care.

## Disclosure

All the authors declared no competing interests.

## Supplementary Material

Supplementary file (PDF) **Figure S1.** Endpoint-free donor survival of groups stratified by presence of predonation arterial hypertension, under the assumption that donors with unclear predonation medical history did not have arterial hypertension, as also no intake of antihypertensive medication was documented. CI, confidence interval ; HR, hazard ratio. **Table S1.** Associations of sex, obesity, age at donation, and baseline eGFR with the longitudinal slope of kidney function after living donation, n = 1863. **Table S2.** Association of predonation hypertension with the longitudinal slope of kidney function after living donation, *n* = 978. **Table S3.** eGFR (ml/min per 1.73 m^2^) category for living kidney donors based on the last available SCr measurement using CKD-EPI equation, *n* = 1863. **Table S4.** Baseline characteristics of donors stratified by reaching the combined endpoint postdonation, n = 1863. **Table S5.** Independent predictors of the combined endpoint postdonation (*n* = 978). Checklist: The STROBE reporting checklist

## Footnotes

Supplementary file (PDF)
**Figure S1.** End point free donor survival of groups stratified by presence of predonation arterial hypertension, under the assumption that donors with unclear predonation medical history did not have arterial hypertension, as also no intake of antihypertensive medication was documented. CI, confidence interval; HR, hazard ratio. **Table S1.** Associations of sex, obesity, age at donation, and baseline eGFR with the longitudinal slope of kidney function after living donation, *n* = 1863. **Table S2.** Association of predonation hypertension with the longitudinal slope of kidney function after living donation, *n* = 978. **Table S3.** eGFR (ml/min per 1.73 m^2^) category for living kidney donors based on the last available SCr measurement using CKD-EPI equation, n = 1863. **Table S4.** Baseline characteristics of donors stratified by reaching the combined end point postdonation, *n* = 1863. **Table S5.** Independent predictors of the combined end point postdonation (*n* = 978). Checklist: The STROBE reporting checklist.

## References

[1] Wolfe R.A., Ashby V.B., Milford E.L. (1999). Comparison of mortality in all patients on dialysis, patients on dialysis awaiting transplantation, and recipients of a first cadaveric transplant. N Engl J Med.

[2] Meier-Kriesche H.U., Port F.K., Ojo A.O. (2000). Effect of waiting time on renal transplant outcome. Kidney Int.

[3] Meier-Kriesche H.U., Kaplan B. (2002). Waiting time on dialysis as the strongest modifiable risk factor for renal transplant outcomes: a paired donor kidney analysis. Transplantation.

[4] Matas A.J., Schnitzler M. (2004). Payment for living donor (vendor) kidneys: a cost-effectiveness analysis. Am J Transplant.

[5] Haller M., Gutjahr G., Kramar R., Harnoncourt F., Oberbauer R. (2011). Cost-effectiveness analysis of renal replacement therapy in Austria. Nephrol Dial Transplant.

[6] Wehmeier C., Georgalis A., Hirt-Minkowski P. (2016). 2222 kidney transplantations at the University Hospital Basel: a story of success and new challenges. Swiss Med Wkly.

[7] Lentine K.L., Smith J.M., Lyden G.R. (2025). OPTN/SRTR 2023 annual data report: kidney. Am J Transplant.

[8] Global Observatory on Donation and Transplantation (GODT) WHO-ONT. https://www.transplant-observatory.org/.

[9] Swiss Transplant Waeteliste und Transplantationen. https://www.swisstransplant.org/.

[10] Reese P.P., Boudville N., Garg A.X. (2015). Living kidney donation: outcomes, ethics, and uncertainty. Lancet.

[11] Lentine K.L., Lam N.N., Axelrod D. (2016). Perioperative complications after living kidney donation: a national study. Am J Transplant.

[12] Burkhalter F., Huynh-Do U., Hadaya K. (2017). Early complications after living donor nephrectomy: analysis of the Swiss Organ Living Donor Health Registry. Swiss Med Wkly.

[13] Garcia-Ochoa C., Feldman L.S., Nguan C. (2019). Perioperative complications during living donor nephrectomy: results from a multicenter cohort study. Can J Kidney Health Dis.

[14] Brugger C., Hunkeler Z., Diebold M. (2024). Early complications in kidney donors and course of health-related quality of life 12 month after donation: an analysis of the swiss organ living-donor health registry. Transplant Direct.

[15] Muzaale A.D., Massie A.B., Wang M.C. (2014). Risk of end-stage renal disease following live kidney donation. JAMA.

[16] Mjoen G., Hallan S., Hartmann A. (2014). Long-term risks for kidney donors. Kidney Int.

[17] Park J.Y., Yang W.J., Doo S.W. (2023). Long-term end-stage renal disease risks after living kidney donation: a systematic review and meta-analysis. BMC Nephrol.

[18] Grams M.E., Garg A.X., Lentine K.L. (2016). Kidney-failure risk projection for the living kidney-donor candidate. N Engl J Med.

[19] Anjum S., Muzaale A.D., Massie A.B. (2016). Patterns of end-stage renal disease caused by diabetes, hypertension, and glomerulonephritis in live kidney donors. Am J Transplant.

[20] Massie A.B., Muzaale A.D., Luo X. (2017). Quantifying postdonation risk of ESRD in living kidney donors. J Am Soc Nephrol.

[21] Lenihan C.R., Busque S., Derby G., Blouch K., Myers B.D., Tan J.C. (2015). Longitudinal study of living kidney donor glomerular dynamics after nephrectomy. J Clin Invest.

[22] Lam N.N., Lloyd A., Lentine K.L. (2020). Changes in kidney function follow living donor nephrectomy. Kidney Int.

[23] Kasiske B.L., Anderson-Haag T.L., Duprez D.A. (2020). A prospective controlled study of metabolic and physiologic effects of kidney donation suggests that donors retain stable kidney function over the first nine years. Kidney Int.

[24] Almeida M., Reis Pereira P., Silvano J. (2024). Longitudinal trajectories of estimated glomerular filtration rate in a European population of living kidney donors. Transpl Int.

[25] Taler S.J., Messersmith E.E., Leichtman A.B. (2013). Demographic, metabolic, and blood pressure characteristics of living kidney donors spanning five decades. Am J Transplant.

[26] Ahmadi A.R., Lafranca J.A., Claessens L.A. (2015). Shifting paradigms in eligibility criteria for live kidney donation: a systematic review. Kidney Int.

[27] Clayton P.A., Saunders J.R., McDonald S.P. (2016). Risk-factor profile of living kidney donors: the Australia and New Zealand dialysis and transplant living kidney donor registry 2004–2012. Transplantation.

[28] Lentine K.L., Kasiske B.L., Levey A.S. (2017). Summary of kidney disease: improving global outcomes (KDIGO) clinical practice guideline on the evaluation and care of living kidney donors. Transplantation.

[29] Welche Entscheidungen für das Schweizer Gsundheitssystem? Diskutieren Sie mit!. https://www.samw.ch.

[30] Boudville N., Prasad G.V., Knoll G. (2006). Meta-analysis: risk for hypertension in living kidney donors. Ann Intern Med.

[31] Garg A.X., Muirhead N., Knoll G. (2006). Proteinuria and reduced kidney function in living kidney donors: a systematic review, meta-analysis, and meta-regression. Kidney Int.

[32] O’Keeffe L.M., Ramond A., Di Angelantonio E. (2018). Midand long-term health risks in living kidney donors. Ann Intern Med.

[33] Ferro C.J., Townend J.N. (2022). Risk for subsequent hypertension and cardiovascular disease after living kidney donation: is it clinically relevant?. Clin Kidney J.

[34] Matas A.J., Rule A.D. (2022). Long-term medical outcomes of living kidney donors. Mayo Clin Proc.

[35] Garg A.X., Prasad G.V., Thiessen-Philbrook H.R. (2008). Cardiovascular disease and hypertension risk in living kidney donors: an analysis of health administrative data in Ontario, Canada. Transplantation.

[36] Muzaale A.D., Massie A.B., Al Ammary F. (2020). Donor-recipient relationship and risk of ESKD in live kidney donors of varied racial groups. Am J Kidney Dis.

[37] Garg A.X., Arnold J.B., Cuerden M.S. (2024). Hypertension and kidney function after living kidney donation. JAMA.

[38] Steiner R.W. (2024). Heeding the increased exponential accumulation of ESRD after living kidney donation. Transplantation.

[39] Thiel G.T., Nolte C., Tsinalis D. (2011). Prospective Swiss cohort study of living-kidney donors: study protocol. BMJ Open.

[40] Krattli J., Buess D., Diebold M. (2025). Metabolic risk after living kidney donation: an analysis of the Swiss Organ Living-Donor Health Registry. Swiss Med Wkly.

[41] Levin A., Ahmed S.B., Carrero J.J. (2024). Executive summary of the KDIGO 2024 Clinical Practice Guideline for the Evaluation and Management of Chronic Kidney Disease: known knowns and known unknowns. Kidney Int.

[42] Ibrahim H.N., Foley R., Tan L. (2009). Long-term consequences of kidney donation. N Engl J Med.

[43] Wainright J., Robinson A., Klassen D. (2018). Living kidney donor risk of ESRD. Am J Transplant.

[44] Kasiske B.L., Anderson-Haag T., Ibrahim H.N. (2013). A prospective controlled study of kidney donors: baseline and 6-month follow-up. Am J Kidney Dis.

[45] Kasiske B.L., Anderson-Haag T., Israni A.K. (2015). A prospective controlled study of living kidney donors: three-year follow-up. Am J Kidney Dis.

[46] Suwelack B., Berger K., Wolters H. (2022). Results of the prospective multicenter SoLKiD cohort study indicate bio-psycho-social outcome risks to kidney donors 12 months after donation. Kidney Int.

[47] Fleetwood V.A., Lam N.N., Lentine K.L. (2025). Long-term risks of living kidney donation: state of the evidence and strategies to resolve knowledge gaps. Annu Rev Med.

[48] Cheng X.S., Glassock R.J., Lentine K.L., Chertow G.M., Tan J.C. (2017). Donation, not disease! A multiple-hit hypothesis on development of post-donation kidney disease. Curr Transplant Rep.

[49] Cortinovis M., Perico N., Ruggenenti P., Remuzzi A., Remuzzi G. (2022). Glomerular hyperfiltration. Nat Rev Nephrol.

[50] Tonneijck L., Muskiet M.H., Smits M.M. (2017). Glomerular hyperfiltration in diabetes: mechanisms, clinical significance, and treatment. J Am Soc Nephrol.

[51] Townsend R.R., Anderson A.H., Chirinos J.A. (2018). Association of pulse wave velocity with chronic kidney disease progression and mortality: findings from the CRIC study (chronic renal insufficiency cohort). Hypertension.

[52] Sanchez O.A., Ferrara L.K., Rein S., Berglund D., Matas A.J., Ibrahim H.N. (2018). Hypertension after kidney donation: incidence, predictors, and correlates. Am J Transplant.

[53] Price A.M., Moody W.E., Stoll V.M. (2021). Cardiovascular effects of unilateral nephrectomy in living kidney donors at 5 years. Hypertension.

[54] Lentine K.L., Schnitzler M.A., Xiao H. (2010). Racial variation in medical outcomes among living kidney donors. N Engl J Med.

[55] Matas A.J., Berglund D.M., Vock D.M., Ibrahim H.N. (2018). Causes and timing of end-stage renal disease after living kidney donation. Am J Transplant.

