# Supplementary material for: Long Term Outcomes in Living Kidney Donors Over 30 Years
Source: Kidney Int Rep. 2026 Jul 2;11(9):106690. doi: 10.1016/j.ekir.2026.106690 (PMC13449912; doi:10.1016/j.ekir.2026.106690)
Supplement: Supplementary file 1 [file mmc1.pdf]

Table S1. Associations of sex, obesity, age at donation, and baseline eGFR with the longitudinal slope of kidney function after living donation, n=1863

Variable	Slope (95% CI)	p-value
- Sex		
- female	0.22 (0.16 to 0.28)	
- male	0.10 (0.02 to 0.18)	
- female vs. male	0.12 (0.03 to 0.21)	0.008
- Obesity at donation (i.e., BMI ≥30 kg/m^2)		
- no	0.20 (0.14 to 0.25)	
- yes	0.04 (-0.1 to 0.18)	
- no vs. yes	0.16 (0.02 to 0.30)	0.03
- Age at donation (yr)		
- 30	0.21 (0.12 to 0.30)	
- 50	0.13 (0.09 to 0.17)	
- 70	0.05 (-0.03 to 0.14)	
- Age 30 yr vs. age 50 yr	0.08 (0 to 0.16)	0.05
- Age 30 yr vs. age 70 yr	0.16 (0 to 0.31)	0.05
- Age 50 yr vs. age 70 yr	0.08 (0 to 0.16)	0.05
- eGFR at donation, CKD-EPI (ml/min/1.73m^2)		
- 60	0.02 (-0.17 to 0.21)	
- 80	0.23 (0.15 to 0.30)	
- 100	0.12 (0.06 to 0.18)	
- eGFR 60 ml/min/1.73m^2 vs. eGFR 80 ml/min/1.73m^2	-0.21 (-0.41 to -0.01)	0.04
- eGFR 60 ml/min/1.73m^2 vs. eGFR 100 ml/min/1.73m^2	-0.10 (-0.3 to 0.10)	0.3
- eGFR 80 ml/min/1.73m^2 vs. eGFR 100 ml/min/1.73m^2	0.10 (0.01 to 0.20)	0.04

Results are derived from a linear mixed-effects model with time treated as a continuous variable. Fixed effects included time and its interactions with sex, obesity at donation, age at donation, and baseline eGFR. Baseline eGFR was modeled using natural cubic splines with three degrees of freedom. Random intercepts and random slopes for time were included at the subject level. Estimates represent adjusted annual eGFR slopes or differences in slopes, with 95% confidence intervals and p-values for the respective comparisons.

Obesity was defined as a BMI ≥30 kg/m^2 according to the WHO definition.

Abbreviations: BMI, body mass index; CI, confidence interval; eGFR, estimated glomerular filtration rate, calculated using the CKD-EPI 2009 equation and expressed in mL/min/1.73 m^2; yr, years.

Table S2. Association of predonation hypertension with the longitudinal slope of kidney function after living donation, n=978

Variable	Slope (95% CI)	p-value
- Predonation hypertension		
- no	0.29 (0.19 to 0.39)	
- yes	0.06 (-0.07 to 0.2)	
- no vs. yes	0.23 (0.06 to 0.4)	0.008

Results are derived from a separate linear mixed-effects model with time treated as a continuous variable. Fixed effects included time, predonation hypertension, the interaction between time and predonation hypertension, and adjustment for sex, obesity at donation, age at donation, and baseline eGFR. Baseline eGFR was modeled using natural cubic splines with three degrees of freedom. Random intercepts and random slopes for time were included at the subject level. Estimates represent adjusted annual eGFR slopes or differences in slopes, with 95% confidence intervals and p-values for the respective comparisons.

Obesity was defined as a BMI ≥30 kg/m² according to the WHO definition.

Abbreviations: BMI, body mass index; CI, confidence interval; eGFR, estimated glomerular filtration rate, calculated using the CKD-EPI 2009 equation and expressed in mL/min/1.73 m².

Table S3. eGFR (ml/min/1.73m^2) category for living kidney donors based on the last available SCr measurement using CKD-EPI equation, n=1863

eGFR (ml/min/1.73m^2)	n (%)
≥90	129 (6.9)
60-89	1048 (56.3)
30-59	669 (35.9)
<30	17 (0.9)

Abbreviations: SCr=serum creatinine, eGFR=estimated glomerular filtration rate (calculated by the CKD-EPI formula; in ml/min/1.73m^2 of body surface). CKD-EPI=Chronic Kidney Disease Epidemiology Collaboration

Table S4. Baseline characteristics of donors stratified by reaching the combined endpoint postdonation, n=1863

Variable	Donors with uneventful postdonation course (n=1823)	Donors reaching the combined endpoint postdonation (n=40)	p-value
- Age at donation (yr)	53 (45-61)	62 (56-65)	**<0.0001**
- Age grouping at donation, n (%)			
- <40 yr	239 (13.1)	3 (7.5)	**<0.0001**
- 40-50 yr	565 (31.0)	1 (2.5)	
- 51-60, yr	592 (32.5)	13 (32.5)	
- 61-70, yr	364 (20.0)	21 (52.5)	
>70 yr	63 (3.4)	2 (5.0)	
- Sex, n (%)			
- Female	1224 (67.1)	9 (22.5)	**<0.0001**
- Male	599 (32.9)	31 (77.5)	
- Donor-recipient relation, n (%)			
- Parents	459 (25.2)	10 (25.0)	0.53
- Siblings	397 (21.8)	13 (32.5)	
- Other relatives	74 (4.0)	1 (2.5)	
- Partners	677 (37.1)	13 (32.5)	
- Other non-relatives	216 (11.9)	3 (7.5)	
- KPD, n (%)	36 (2.0)	0 (0)	1.0
- Era of donation			
- 1993-2000, n (%)	322 (17.7)	19 (47.5)	**<0.0001**
- 2001-2009, n (%)	704 (38.6)	14 (35.0)	
- 2010-2018, n %)	797 (43.7)	7 (17.5)	
- Preexisting diseases, n (%)$^{\Delta}$	723 (39.7)	24 (60.0)	**0.01**
- Hypertension before donation, n (%)	274 (15.0)	14 (35)	**0.001**
- Cardio-vascular diseases, n (%)	44 (2.4)	1 (2.5)	0.94
- Dyslipidemia predonation, n (%)	88 (4.8)	4 (10.0)	0.13
- T2DM before donation, n (%)	7 (0.4)	0 (0)	0.58
- Other diseases predonation	368 (20.2)	7 (17.5)	0.76
- Any medication taken, yes, n (%)	1172 (64.3)	21 (52.5)	0.14
- Smoking, yes, n (%)*	120 (6.6)	4 (10.0)	0.58
- Kidney function before donation			
- Creatinine (µmol/l)	66 (58-77)	80 (63-90)	**<0.0001**
- eGFR, CKD-EPI (ml/min/1.73m^2)	96 (86-105)	90 (79-99)	**0.008**
- eGFR category, CKD-EPI (ml/min/1.73m^2)			
- ≥90, n (%)	1202 (66.9)	20 (50.0)	**0.009**
- 80-89, n (%)	308 (17.1)	9 (22.5)	
- 70-79, n (%)	184 (10.3)	4 (10.0)	
- 60-69, n (%)	87 (4.8)	5 (12.5)	
- 50-59, n (%)	16 (0.9)	2 (5.0)	
- UPCR (mg/mmol)§	7.7 (6.0-10.7)	8.3 (5.5-13.8)	0.28
- UACR (mg/mmol)§	0.6 (0.4-1.0)	1.1 (0.5-4.8)	**0.001**
- Proteinuria category$^{\blacktriangle}$			
- None/mild, n (%)	1215 (88.7)	21 (70.0)	**<0.0001**
- Moderate, n (%)	155 (11.3)	8 (26.7)	
- Severe, n (%)	0 (0.0)	1 (3.3)	
- Systolic blood pressure (mmHg)	126 (118-135)	134 (124-147)	**0.0002**
- Diastolic blood pressure (mmHg)	78 (71-83)	85 (76-90)	**0.0001**
- Body weight (kg)	70 (62-80)	78 (71-87)	**<0.0001**
- BMI (kg/m^2)	25.0 (22.7-27.7)	26.9 (24.9-28.3)	**0.004**
- <25 kg/m^2	907 (50)	10 (25)	**0.006**
- 25-29.9 kg/m^2	710 (39.2)	22 (55)	
- 30-34.9 kg/m^2	173 (9.5)	8 (20)	
- ≥35 kg/m^2	24 (1.3)	0 (0)	

Values are median (IQR) unless otherwise stated. Abbreviations: yr=years, KPD=kidney paired donation, eGFR=estimated glomerular filtration rate (calculated by the CKD-EPI 2009 formula; in ml/min/1.73m^2 of body surface). CKD-EPI=Chronic Kidney Disease Epidemiology Collaboration, #eGFR was missing for n=26 donors. §UPCR=urine protein-to-creatinine ratio and §UACR=urine albumin-to-creatinine ratio, which was only calculated for undiluted urine samples (i.e., urine creatinine ≥ 3 mmol/l; n=1400). ▲Proteinuria was defined by UPCR or UACR based on the Kidney Disease Improving Global Outcomes (KDIGO) definitions as none/mild (UPCR<15 mg/mmol, or UACR <3 mg/mmol), moderate (UPCR 15-50 mg/mmol, UACR 3-30 mg/mmol), and severe (UPCR >50 mg/mmol, UACR >30mg/mmol). BMI=Body mass index (in total 1854 values were available).

[Δ]In total, 747 (40.1%) living kidney donors had one or more preexisting diseases.

*Smoking behavior is known of n=838 (45.0%) living kidney donors.

Table S5. Independent predictors of the combined endpoint postdonation (n=978)

	Donors with unexpected postdonation course (n=956)	Donors reaching the combined endpoint postdonation (n=22)	Univariate Cox proportional analysis Hazard ratio (95% CI); p-value	Multivariable Cox proportional analysis# Hazard ratio (95% CI); p-value
**Predictors of combined endpoint**				
- Era of donation, n				
- 2001-2009 vs. 1993-2000, n	218/51	9/8	2.73 (0.66-11.26); 0.17	2.93 (0.70-12.3); 0.14
- 2010-2018 vs. 1993-2000, n	687/51	5/8	2.24 (0.350-14.48); 0.40	3.18 (0.48-21.0); 0.23
- 2010-2018 vs. 2001-2009, n	687/218	5/9	0.82 (0.21-3.19); 0.77	1.08 (0.27-4.25); 0.91
- Predonation obesity (BMI≥30 kg/m^2)*				
- yes vs no, n (%)	126 (13.2)/830 (86.8)	5 (22.7)/17 (77.3)	2.27 (0.82-6.27); 0.11	2.02 (0.72-5.64); 0.18
Predonation arterial hypertension				
- yes vs no, n (%)	274 (28.7)/682 (71.3)	14 (63.6)/8 (36.4)	3.53 (1.41-8.83); **0.007**	3.42 (1.34-8.68); **0.01**

Abbreviations: CI= Confidence interval, yr=years, BMI=Body mass index (kg/m^2)

*Predonation BMI was available of n=1857 donors.

#The last column represents the whole multivariable model (analyzed with n=978 donors).

Figure S1

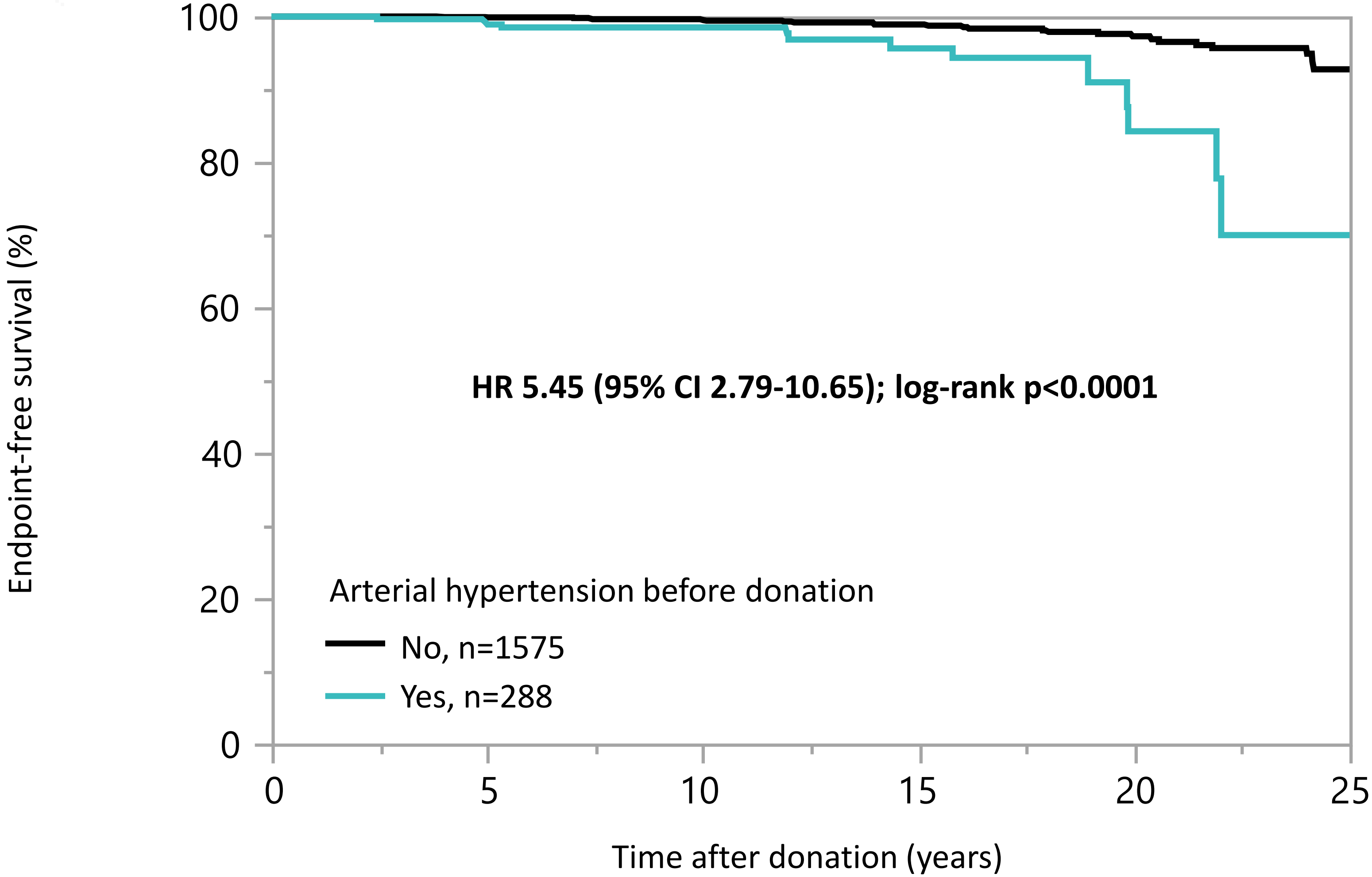

Donors at risk (n)											
No (black)	1575	1575	1495	1113	1044	762	594	448	303	151	81
Yes (teal)	288	287	265	171	146	99	74	45	20	8	3

Figure S1. Endpoint-free donor survival of groups stratified by presence of predonation arterial hypertension, under the assumption that donors with unclear predonation medical history did not have arterial hypertension, as also no intake of antihypertensive medication was documented. HR=hazard ratio, CI=confidence interval

The STROBE reporting checklist

For checking that observational epidemiology research articles can be understood and used by everyone

> Note
>
> If you have not used a reporting guideline before, read about how and why to use them and check whether STROBE is the most applicable reporting guideline for your work.
>
> Reporting guidelines are most useful when used early in research. When writing a manuscript or application, consider using the Full Guidance where you'll see explanations and examples for each item.
>
> After writing, demonstrate adherence by completing this checklist:
>
> 1. Specify where each item is described (see Note 1).
> 2. Cite this checklist (See Note 2).
> 3. Include your completed checklist as a supplement when submitting to a journal so that future readers can use it to find information.

	Item Description	Location (or reason for not reporting)
**Title and abstract**		
1a. Indicate the study's design	Indicate the study's design with a commonly used term in the title or the abstract.	p. 5, Abstract
1b. Abstract	Provide in the abstract an informative and balanced summary of what was done and what was found.	p. 4, Abstract
**Introduction**		
2. Background / rationale	Explain the scientific background and rationale for the investigation being reported.	pp. 6-7, Introduction
3. Objectives	State specific objectives, including any prespecified hypotheses.	p. 7, end of Introduction section
**Methods**		
4. Study design	Present key elements of study design early in the paper.	pp. 7-8, Methods
5. Setting	Describe the setting, locations, and relevant dates, including periods of recruitment, exposure, follow-up, and data collection.	pp. 7-8, Methods (Donor population, Data collection)
6a. Eligibility criteria	**Cohort study:** Give the eligibility criteria, and the sources and methods of selection of participants. Describe methods of follow-up. **Case-control study:** Give the eligibility criteria, and the sources and	pp. 7-8, Methods (Donor population)

	methods of case ascertainment and control selection. Give the rationale for the choice of cases and controls. **Cross-sectional study:** Give the eligibility criteria, and the sources and methods of selection of participants.	
6b. Matching criteria	**Cohort study:** For matched studies, give matching criteria and number of exposed and unexposed. **Case-control study:** For matched studies, give matching criteria and the number of controls per case.	N/A
7. Variables	Clearly define all outcomes, exposures, predictors, potential confounders, and effect modifiers. Give diagnostic criteria, if applicable.	pp. 9-10, Methods
8. Data sources / measurement	For each variable of interest give sources of data and details of methods of assessment (measurement). Describe comparability of assessment methods if there is more than one group.	pp. 8-9, Methods (Data collection and Table 2)
9. Bias	Describe any efforts to address potential sources of bias.	p. 10, Methods
10. Study size	Explain how the study size was arrived at.	pp. 7-8, Methods
11. Quantitative variables	Explain how quantitative variables were handled in the analyses. If applicable, describe which groupings were chosen, and why.	p. 10, Methods
12a. Statistical methods	Describe all statistical methods, including those used to control for confounding.	p. 10, Methods
12b. Statistical methods – subgroups and interactions	Describe any methods used to examine subgroups and interactions.	p. 10, Methods
12c. Statistical methods – missing data	Explain how missing data were addressed.	p. 8, Methods
12di. Statistical methods – loss to follow-up	**Cohort study:** If applicable, describe how loss to follow-up was addressed.	N/A
12dii. Statistical methods – matching cases and controls	**Case-control study:** If applicable, explain how matching of cases and controls was addressed.	N/A
12diii. Statistical methods – sampling strategy	**Cross-sectional study:** If applicable, describe analytical methods taking account of sampling strategy.	p. 10, Methods
12e. Statistical methods – sensitivity analyses	Describe any sensitivity analyses.	p. 10, Methods

**Results**		
13a. Participant numbers	Report the numbers of individuals at each stage of the study—e.g., numbers potentially eligible, examined for eligibility, confirmed eligible, included in the study, completing follow-up, and analysed; Consider use of a flow diagram.	Figure 1, Table 2
13b. Participants – non-participation	Give reasons for non-participation at each stage.	N/A
13c. Participants – flow diagram	Consider use of a flow diagram.	Figure 1
14a. Descriptive data – participant characteristics	Give characteristics of study participants (e.g., demographic, clinical, social) and information on exposures and potential confounders. Present the information in a table.	Table 1, description pp. 11-12, Results
14b. Descriptive data – missing data	Indicate the number of participants with missing data for each variable of interest.	Table 1-6
14c. Descriptive data – follow-up time	**Cohort study:** Summarise follow-up time—e.g., average and total amount.	p.12, Results
15. Outcome data	**Cohort study:** Report numbers of outcome events or summary measures over time. **Case-control study:** Report numbers in each exposure category, or summary measures of exposure. **Cross-sectional study:** Report numbers of outcome events or summary measures.	Figure 2, Figure 3A-C and Figure 4A-D, description see p. 12-15, Results
16a. Main results	Give unadjusted estimates and, if applicable, confounder-adjusted estimates and their precision (e.g., 95% confidence intervals). Make clear which confounders were adjusted for and why they were included.	Table S1, S2
16b. Main results – category boundaries	Report category boundaries when continuous variables were categorised.	Table 2, Table 3, Table S3
16c. Main results – risk	If relevant, consider translating estimates of relative risk into absolute risk for a meaningful time period.	N/A
17. Other analyses	Report other analyses done—e.g., analyses of subgroups and interactions, and sensitivity analyses.	p. 15, Results, Figure S1
**Discussion**		
18. Key results	Summarise key results with reference to study objectives.	p. 16-19, Discussion
19. Limitations	Discuss limitations of the study, taking into account sources of potential bias or imprecision. Discuss both direction and magnitude of any potential bias.	p. 19, Discussion

20. Interpretation	Give a cautious overall interpretation considering objectives, limitations, multiplicity of analyses, results from similar studies, and other relevant evidence.	pp. 16-19, Discussion
21. Generalisability	Discuss the generalisability (external validity) of the study results.	p. 19, Discussion
**Other information**		
22. Funding	Give the source of funding and the role of the funders for the present study and, if applicable, for the original study on which the present article is based.	p. 21, description of funding

1 How to specify where content is

Tell the reader where they can find information. E.g.,

- Results; paragraph 2
- Methods, Participants; paragraphs 1 & 2.
- Table 3
- Supplement B, para. 4

If you have chosen not to describe an item, explain why. You can do this in the checklist, or as a note below it.

You can describe items in the article body, or in tables, figures, or supplementary materials, and should prioritize items you feel are most important to your intended audience. The order of items in your manuscript does not need to match the order of items in this checklist. You can decide how best to structure your work.

2 How to cite

Describe how you used STROBE at the end of your Methods section, referencing the resources you used e.g.,

> 'We used the STROBE reporting guideline(1) to draft this manuscript, and the STROBE reporting checklist(2) when editing, included in supplement A'

If you use a reporting checklist, remember to include it as a supplement when publishing so that readers can easily find information and see how you have interpreted the guidance.

1. Elm E von, Altman DG, Egger M, Pocock SJ, Gøtzsche PC, Vandenbroucke JP, et al. The strengthening the reporting of observational studies in epidemiology (STROBE) statement: Guidelines for reporting observational studies. Annals of Internal Medicine [Internet]. 2007 Oct;147(8):573–7. Available from: https://www.acpjournals.org/doi/10.7326/0003-4819-147-8-200710160-00010

2. Elm E von, Altman DG, Egger M, Pocock SJ, Gøtzsche PC, Vandenbroucke JP, et al. The STROBE reporting checklist. In: Harwood J, Albury C, Beyer J de, Schlüssel M, Collins G, editors. The EQUATOR network reporting guideline platform [Internet]. The UK EQUATOR Centre; 2025. Available from: https:/resources.equator-network.org/reporting-guidelines/strobe/strobe-checklist.docx
